# *AprioriGWAS*, a New Pattern Mining Strategy for Detecting Genetic Variants Associated with Disease through Interaction Effects

**DOI:** 10.1371/journal.pcbi.1003627

**Published:** 2014-06-05

**Authors:** Qingrun Zhang, Quan Long, Jurg Ott

**Affiliations:** 1 Department of Genetics and Genomic Sciences, Institute of Genomics and Multi-scale Biology, Icahn School of Medicine at Mount Sinai, New York, New York, United States of America; 2 Institute of Psychology, Chinese Academy of Sciences, Chaoyang District, Beijing, PR China; 3 Laboratory of Statistical Genetics, The Rockefeller University, New York, New York, United States of America; University of Chicago, United States of America

## Abstract

Identifying gene-gene interaction is a hot topic in genome wide association studies. Two fundamental challenges are: (1) how to smartly identify combinations of variants that may be associated with the trait from astronomical number of all possible combinations; and (2) how to test epistatic interaction when all potential combinations are available. We developed *AprioriGWAS*, which brings two innovations. (1) Based on *Apriori*, a successful method in field of Frequent Itemset Mining (FIM) in which a pattern growth strategy is leveraged to effectively and accurately reduce search space, *AprioriGWAS* can efficiently identify genetically associated genotype patterns. (2) To test the hypotheses of epistasis, we adopt a new conditional permutation procedure to obtain reliable statistical inference of Pearson's chi-square test for the 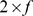 contingency table generated by associated variants. By applying *AprioriGWAS* to age-related macular degeneration (AMD) data, we found that: (1) angiopoietin 1 (ANGPT1) and four retinal genes interact with Complement Factor H (CFH). (2) GO term “glycosaminoglycan biosynthetic process” was enriched in AMD interacting genes. The epistatic interactions newly found by *AprioriGWAS* on AMD data are likely true interactions, since genes interacting with CFH are retinal genes, and GO term enrichment also verified that interaction between glycosaminoglycans (GAGs) and CFH plays an important role in disease pathology of AMD. By applying AprioriGWAS on Bipolar disorder in WTCCC data, we found variants without marginal effect show significant interactions. For example, multiple-SNP genotype patterns inside gene GABRB2 and GRIA1 (AMPA subunit 1 receptor gene). AMPARs are found in many parts of the brain and are the most commonly found receptor in the nervous system. The GABRB2 mediates the fastest inhibitory synaptic transmission in the central nervous system. GRIA1 and GABRB2 are relevant to mental disorders supported by multiple evidences.

## Author Summary

Genes do not operate in vacuum. They interact with each other in many ways. Therefore, to figure out genetic causes of disease by case-control association studies, it is important to take interactions into account. There are two fundamental challenges in interaction-focused analysis. The first is the number of possible combinations of genetic variants easily goes to astronomic which is beyond current computational facility, which is referred as “the curse of dimensionality” in field of computer science. The other is, even if all potential combinations could be exhaustively checked, genuine signals are likely to be buried by false positives that are composed of single variant with large main effect and some other irrelevant variant. In this work, we propose *AprioriGWAS* that employees *Apriori*, an algorithm that pioneers the branch of “Frequent Itemset Mining” in computer science to cope with daunting numbers of combinations, and *conditional permutation*, to enable real signals standing out. By applying *AprioriGWAS* to age-related macular degeneration (AMD) data and bipolar disorder (BD) in WTCCC data, we found interesting interactions between sensible genes in terms of disease. Consequently, *AprioriGWAS* could be a good tool to find epistasis interaction from GWA data.

## Introduction

Gene-gene interactions have been proposed as one potential explanation of the well-known problem of missing heritability [1], and a recent report [2] has quantitatively demonstrated that possibility. Researchers have long attempted to identify interactions, with methods ranging from evolutionary genetic studies [3], [4], systems biology studies of model microbes [5] and quantitative genetic studies of inbred model organisms, to linkage [6] and association studies in human populations [7]–[14]. Although the definitions of the term “epistasis” used by biologists (Batson 1909) [15] and statisticians (Fisher 1918) [16] are different, they have the same consequences regarding different distributions of genotype patterns among different phenotypes.

The main obstacle of interaction analysis is that the large number of multi-locus genotype combinations generated from large numbers of genetic variants is too high for current computational resources. This is in fact a well-known computational problem, known in the field of computer science as the ‘curse of dimensionality’ [17]. In this work we developed *AprioriGWAS*, a tool to address this problem. This tool is based on a successful algorithm in the field of computer science, *Apriori* [18].

*Apriori* was originally designed for supermarket data mining to assist shop owners in designing the layout of displayed products. Given customers' transactions, the algorithm can identify sets of items that frequently co-exist in transactions. For example, by knowing that customers usually buy milk and bread together, the shop owner can put them near each other in the store.

Before describing the algorithm, we briefly give definitions of a few key terms: *item* is defined as an individual product, for example, bought by a customer; *itemset* stands for a set of items purchased together; *length* of itemset is defined as the number of items in the itemset. The process of growing a short itemset to a longer itemset is referred to as pattern growth. Generally, the key insights of *Apriori* are that: (1) frequent itemset with many items can be gained by growing itemset of short length; and (2) since subsets of any frequent itemset should also be frequent during pattern growth, itemsets predicted not to have any effect can be dropped during pattern growth, thereby significantly reducing the search space. In the case of GWAS, the number of individual genotypes is analogous to the number of transactions in supermarket data. The genotype of a variant is an item, and genotype combinations of different variants are an itemset, here also called a genotype pattern. Instead of just finding frequent genotype patterns, we want to find genotype patterns with different frequencies in cases and controls. We call them differential genotype patterns. While *Apriori* originally works on one database to find the most frequent itemsets, we are interested in patterns with different frequencies in two databases (cases and controls). To assess whether a pattern should be retained during pattern growth, we make use of the *proportion test* [19] (**Methods**).

Interaction among variants is carried out after obtaining all differential genotype patterns. We test the possibility of interaction among variants involved in a differential genotype pattern by conducting Pearson's Chi-square test for the contingency tables composed of all genotype patterns found for variants and phenotypes (**Methods**). In this step, we try to distinguish whether a differential pattern is caused by variants with marginal effects or by interaction effect. The process of pattern growth helps to narrow down the number of variant combinations to be tested for interaction effect.

Using simulations following Marchini *et al's* procedure [11], we demonstrate that *AprioriGWAS* can approximately achieve the same coverage of associated patterns as an exhaustive search, but with far lower CPU time.

Determining all potential combinations that are statistically associated with disease does not automatically identify genuinely interacting genes. The daunting number of all combinations of variants heavily increases the load of multiple tests and mixes genuine signals with noise. As summarized by Anderson [20], in the regression model with two main effects terms and one interaction term, there is no exact permutation method for testing the significance of the interaction term. Buzkova *et al* [21] proposed a parametric bootstrap test for gene-gene and gene-environment interactions, which unfortunately is not practical for very large numbers of possible combinations of variants. Computer simulation [22] shows that whenever a trait is controlled by more than a single factor, it becomes possible for a neutral variant together with a major-effect variant as a pattern to be more strongly associated with the trait than with any of the causative factors [13]. These indirect associations are true associations for statistical purposes, and can be indistinguishable from medical causative associations [22]. To distinguish general association and interaction effects, we developed a new *conditional permutation* test to distinguish genuine interactions from the artifacts generated by the combination of a major-effect variant with a neutral variant (**Methods**). We demonstrate that our new approach has a magnitude lower false discovery rate (FDR) compared with regular permutation, while maintaining comparable power.

We applied *AprioriGWAS* to age-related macular degeneration (AMD [MIM 153800]), which has been deemed a good example of a small number of common variants explaining a large proportion of heritability [1]. Among the most significant patterns, we found six pairs of retinal genes interacting with each other. An exciting example is the interaction of a gene involved in an AMD treatment target, ANGPT1, with another important AMD gene, CFH. Overall, the potentially interacting genes were enriched in glycosaminoglycan biosynthetic process (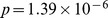). Many studies have shown that the interaction between glycosaminoglycans (GAGs) and CFH plays an important role in the disease pathology of AMD. We also applied *AprioriGWAS* to bipolar disorder; we found potential interactions inside individual gene (8 out of 18 genes are related with mental disorder) and interactions across gene or chromosomes. Further results will be presented in full later.

The remainder of this paper is organized as follows. In the next section we introduce the *AprioriGWAS* algorithm for mining possible interaction variants, as well as the conditional permutation approach for testing interactions. We then evaluate the performance of *AprioriGWAS* with simulated data and compare it with logistic regression implement in Epistasis function of PLINK. Lastly we demonstrate applications of *AprioriGWAS* to AMD and WTCCC bipolar data and exciting findings from both datasets.

## Materials and Methods

### Original *Apriori* Algorithm

Historically, the *Apriori* algorithm can be traced back to the seminal paper published by IBM Research in 1993 [18]. The concept of the main technique is that a subset of frequent itemset should also be frequent. Based on this concept, frequent itemset with more items may be found by stepwise growth of smaller frequent itemset, which saves substantial computational resources. Interested readers may refer to their original paper [18] for a professional description or to our own longer report [23] for illustrative descriptions. Here we briefly outline the main steps. Suppose one wants to mine frequent itemset with length no more than *n*. *Apriori* will usually scan dataset in *n* rounds (unless there is no new frequent itemset generated in a certain round before *n*, thereby forcing the algorithm to halt). In the first round, it will initiate the 1-itemsets that are frequent. In each subsequent round, it will take the frequent itemset generated in the last round as starting point and grow any itemset by adding one more item. Retention of the new itemset will be decided by firstly predicting how likely it will be and then, given a positive prediction, by checking the actually supporting transactions. Finally, the collection of all frequent itemset in all rounds will be reported.

### Algorithm of *AprioriGWAS*

In this paper, genotype patterns are defined as genotype combinations of different variants. We use integer numbers as ids of variants; then we can have, for instance, a pattern like 46**_AT_**_609**_GG_**_1099_CC_, denoting a pattern composed of a variant with id 46 and genotype AT combined, a variant with id 609 and genotype GG, and a variant with id 1099 and genotype CC. The key goal is to find genotype patterns that have a significant frequency difference in cases and controls (called *differential* patterns in this paper).

The algorithm of *AprioriGWAS* is divided into two steps. First, detecting differential genotype patterns by an *Apriori*-like strategy. Obviously, the same set of variants can lead to several differential genotype patterns. Second, testing interaction among a set of variants by testing association of all possible combinations of genotype patterns against case/control status. The first step helps to narrow down the combinations of variants need to be tested. Due to multiple test problems and potential association of single variants involved in the differential genotype pattern, we adopt a new conditional permutation in the second step to control the marginal effect of single variants for testing of variant interactions.

#### 1) Detecting differential genotype patterns

The first step of *AprioriGWAS* generally follows the flow of the original *Apriori* described above. Genotype patterns start from one single genotype, then, in each subsequent round, genotype pattern growth occurs by adding one more genotype of a new variant. Explicitly, for a given pattern length, we scan each pattern in the candidate set against all candidate genotypes of the remaining variants to see whether a variant should be included. The main difference is that the criteria of predicting whether the growth of a pattern should be retained is replaced by a proportion test [19] that fits the scenario of case/control studies.

Essentially, the proportion test is to test whether a genotype pattern has the same frequency between cases and controls (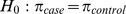 *vs* 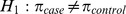). We denote the genotype frequency in the union of cases and controls by *π*. In the following equation, 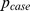, 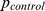 and 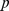 are respective estimates of 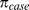, 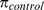 and 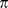. Then we have
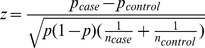
Under the null hypothesis of no difference in frequencies, the square of the statistic, 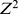 follows a chi-square distribution with one degree of freedom.

For a pattern potentially being significant, it must contain sub-patterns with moderate marginal effect to pass the proportion test at the initial round. However, it is possible that some sub-patterns with no marginal effect could contribute to interacting modules [24]. (In particular, single variant can be deemed as a pattern with size 1.) We thus face a trade-off here: too strict a threshold of the proportion test will exclude potential patterns that contain sub-patterns without marginal effect, whereas too relaxed threshold will end up with too many candidates to test. To balance this trade-off, we add *ad hoc* criteria for pattern growth. The idea is that we assume that the genotype pattern with more than one variant without marginal effect is not likely to be useful, while the pattern with just one variant is more likely to be. For example, for a pattern with length = 3, if all of its 2-item sub-pattern are not significant, we assume that this pattern cannot be significant and will remove it from the candidates; however, if all of its 1-item sub-pattern are not significant, we still retain this pattern as potential target. Formally, when pattern length is not greater than one, both differential patterns and non-differential patterns with relatively high frequency will be kept in the candidate set for pattern growth. When pattern length exceeds one, only differential patterns will be used in the next round of pattern growth.

#### 2) Testing genotype pattern association against case/control status

After obtaining a list of differential genotype patterns by the innovative pattern-growth algorithm, we generate a 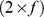 contingency table for variants involved in differential genotype patterns. The two columns stand for cases and controls; 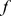 rows are for the genotype patterns composed of the involved variants to be tested. To prevent potential problems of a sparse contingency table, we aggregate genotype patterns rare in both cases and controls into one group. We thereby have 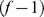 major patterns, plus an extra row of rare patterns. Then we assess the global deviation from randomness by Pearson's chi-square test with 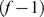 degrees of freedom.

#### 3) Control family-wise error rate by conditional permutation

In genome wide association studies, more than 100,000 variants are generally tested. For gene-gene interaction studies, the possible combination for testing is even higher. With large numbers of tests being carried out, we need to correct for multiple testing to keep the global significance level under control. Various solutions have been demonstrated on published data. Permutation tests are widely used in genomic studies. However, as it has been summarized by Anderson [25] and further investigated by Buzkova *et al* [21], both regular permutation and traditional conditional permutation are not valid to test gene-gene or gene-environment interactions. Before proposing our new development, and to keep the paper self-contained, we summarize their insight as follows. We first consider a test for interaction between the effects of a single genetic variant and an environmental exposure E on a phenotype Y. (E could be another genetic variant), as described by:
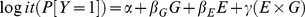
(1)The null hypothesis is that the interaction term has no effect 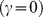 while *G* and *E* may have effects. To test whether 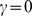, a regular permutation test would permute all outcomes 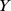 to give 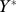. In the permuted dataset, 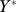 is independent of *G* and *E* and 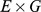. However, in equation (1), it is not necessary that *Y* be independent of *G* and *E*. Buzkova *et al*'s simulation showed that regular permutation is not valid to test interaction in such a situation. On the other hand, for the null hypothesis of one categorical main effect (e.g. E has an effect on the outcome of *Y*), one may be interested in comparing the null hypothesis of 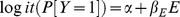 to the full alternative (1), testing 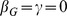. Traditional conditional permutation, which permutes *Y* within individual strata of *E*, is not valid for specifically testing no interaction (Anderson [20]). Thus, Buzkova *et al* [21] proposed a parametric bootstrap test for gene-gene and gene-environment interactions. In principle, the authors fix *G* and *E* and generate 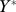 for each individual as a binary variable satisfying
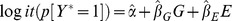
Where 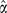 and 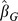, 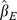 are estimated from the original data under the null model of (1). Then the authors compute the test statistic for the simulated sample and repeat the process many times to obtain the test statistic's distribution under the null hypothesis. Correspondingly, the significance level of an observed value could be evaluated from simulated test statistics. Applying the parametric bootstrap strategy for all pairs of candidates would be computationally unaffordable for whole genome analysis. In addition, the main effect of individual variants will be removed in a regression model. However, for Pearson's Chi-Squared test of the contingency table, the main effect of individual variants and their interaction effect are mixed. We therefore propose a new conditional permutation strategy below.

*Test statistics and null hypothesis*. As described above, we get a 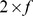 contingency table for variants involved in differential genotype patterns, and then do a Pearson's Chi-Squared test for the 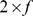 contingency table. Our test statistic is the p-value of the Pearson's Chi-Squared test of the contingency table.

The null hypothesis H_0_ is that, conditional on the individual main effect of the variant with highest marginal effect (higher than that of all other variants in the pattern), there is no extra interaction among the variants that contribute to the association level. Precisely speaking, for any significance level, 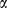, of marginal effect, 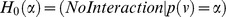, where 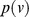 denotes the p-value of variant *v* in single marker test, and *v* has the highest marginal effect compared with other involved variants in the pattern.

To test whether 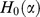 holds, our test statistic is the p-value of Pearson's Chi-Squared test of the contingency table composed of all variants in the pattern, conditional on the p-value of *v*, 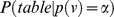, and generate its null distribution using the conditional permutation described below.

*A modified conditional permutation*. Formally, the procedure is as follows: assuming variant *v* has the strongest marginal effect among the variants involved in a given differential pattern, we retain the association of *v* with the phenotype outcome 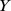 (i.e., when the labels of individuals change, 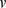 will change accordingly), and permute 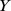 to yield 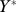. By this permutation, 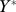 is independent of all other variants, but keeps its dependency with *v*. This permutation thereby yields the null distribution of the p-value of Pearson's Chi-Squared test of the contingency table when a main effect of *v* is present.

More precisely: we use *N* to denote the vectors composed of 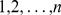 where *n* is the sample size, and use *m* to denote the number of variants. A permutation is denoted by a mapping 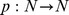. Suppose the phenotype and genotype data before permutation are
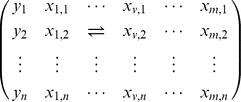
Then after permutation it may be:
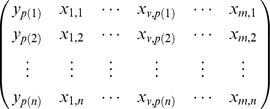
As in the standard procedure, for each permutated dataset, we repeat the whole process of mining patterns, getting the smallest p-value for the contingency table. By generating many permuted datasets, the empirical distribution of test statistics under null hypothesis is obtained. Correspondingly, the p-values in observed data are calculated as the proportions of permuted test statistics that are at least as extreme as the observed value.

As explained above, performing parametric bootstrap tests for each pair of variants would be computationally expensive; now the same problem applies to our initial strategy. To make the test of interaction feasible for GWAS data, some computational tricks have to be employed. Since different variants with the same significance level of marginal effect will share the same null distribution, it is feasible to calculate the null distributions in advance and use that for each variant. We therefore choose to group variants with a similar level of marginal effects and use the same threshold for each group. For example, variants with p-value between (0.001∼0.0001) in the single marker test will use the same threshold calculated in advance from the null distribution. We thereby obtain the table of thresholds for patterns composed of variants with different categories of marginal effect and make use of them as a lookup table during the analysis.

Formally, we calculate the table of thresholds as follows. For variants with p-values in the single marker test of less than 0.001, we set one threshold for each order of magnitude (i.e., from 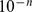 to 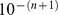, where 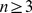 *n*≥3). For all other variants with p-value larger than 0.001 (e.g, 0.1), we treat them as one extra group. For each group of variants we choose the lower limit of the p-value to do conditional permutation. For example, in the analysis of AMD data, among 103,611variants, 62 variants have p-values within (0.001, 0.0001), 8 variants have p-values in (10^−*4*^, 10^−5^), one variant has a p-value in (10^−5^, 10^−6^), one variant has a p-value in (10^−*6*^, 10^−7^), and all others have p-values exceeding 10^−*3*^. We choose the most significant one in each class, and do a conditional permutation for that variant, thereby obtaining the critical value for the contingency table composed of variants no more significant than the lower limit of each class. We then compare results from the non-permuted dataset with the pre-calculated lookup table to obtain the significant combinations of variants.

### Effect Models in Data Simulation

#### Theoretical two-locus interaction models

To make our methods more comparable with existing methods, we adapt Marchini *et al*'s [11] two-locus interaction models. To keep the paper self-contained, we briefly describe the procedure here. **Table 1** **–** [Table pcbi-1003627-t002] **3** describes three two-locus interaction models. Capitalized letters denote the disease allele. In Model 1, the odds of disease increase multiplicatively with genotype both within and between loci. With increasing numbers of the disease allele in a genotype, odds of having the disease increase multiplicatively. The odd of disease for the genotype combination at two interacting loci is the product of the two within-locus effects. Model 2 and 3 require that both loci have at least one copy of the disease associated allele for the odds to increase beyond the baseline level. The difference is that in Model 2 each additional copy of the disease-associated allele further increases the odds by a multiplicative factor, whereas in Model 3, additional copies of disease-associated alleles do not further increase the risk.

**Table 1:** Model 1: Multiplicative effects within and between loci model.

	*bb*	*Bb*	*BB*
aa	*α*	*α (1+θ)*	*α (1+θ)^2^*
Aa	*α(1+θ)*	*α (1+θ)^2^*	*α (1+θ)^3^*
AA	*α(1+θ)^2^*	*α (1+θ)^3^*	*α (1+θ)^4^*

Theoretical models (adopted from Marchini *et al* [11]) for data simulations.

**Table 2 pcbi-1003627-t002:** Model 2: Threshold effects then multiplicative effects model.

	*bb*	*Bb*	*BB*
aa	*α*	*α*	*α*
Aa	*α*	*α (1+θ)*	*α (1+θ)^2^*
AA	*α*	*α (1+θ)^2^*	*α (1+θ)^4^*

Theoretical models (adopted from Marchini *et al* [11]) for data simulations.

**Table 3:** Model 3: Threshold effects with no multiplicative effects model.

	*bb*	*Bb*	*BB*
aa	*α*	*α*	*α*
Aa	*α*	*α (1+θ)*	*α (1+θ)*
AA	*α*	*α (1+θ)*	*α (1+θ)*

Theoretical models (adopted from Marchini *et al* [11]) for data simulations.

For power simulations, we adopt all the parameters (allele frequencies 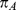 and 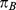, prevalence of disease *p* and parameter 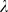) and definitions from Marchini *et al*'s work [11]. For more details, please see supplements of their paper [11]. To make this paper self-contained, the definition of these parameters are listed below:
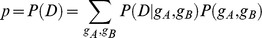


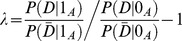
We set disease prevalence to 0.1, parameter *λ* ranges from 0.2, 0.3, 0.5 to 0.7, and the two interacting loci (A and B) have population allele frequencies 0.05, 0.1, 0.2, and 0.5.

#### Epistasis models in classical textbook simulated based on HapMap genotype

In addition to the theoretical interaction models heavily used in the literatures that aim to develop new statistical methods for gene interactions, we also simulate phenotype practically studied interaction models using real genotypes.

In classical textbooks on genetics, a technique to detect epistasis (usually in animal or plant breeding practice) is to check whether the proportions in an F2 population fit theoretical predictions of hypothetical interaction type (**Table 4**). Although that are practices in breeding studies instead of human studies, the well-studied models still serve as established genetic template for simulations that may be closer to real traits. Among the six classical models, there are three models, i.e., *Duplicate dominant*, *Duplicate recessive*, and *Dominant & recessive interaction*, that contain two distinct phenotype values (in contrast to the other three with more than two values) (**Table 4**). Here, as described in standard textbooks, the term “*Duplicate dominant*” denotes the scenario that a single mutated allele in any of the two focal genes will cause phenotypic change; “*Duplicated recessiv*e” denotes the case that a homozygote genotype in any of the two focal genes will cause phenotypic change; “*Dominant & recessive interaction*” denotes the events that either a single mutated allele in the first focal gene or no mutated allele in the first gene together with a mutated allele in the second gene will lead phenotypic change. We deem these two phenotype values as indicators of case or control and simulate phenotype based on real genotype from unrelated CEU samples of HapMap III (http://hapmap.ncbi.nlm.nih.gov) (sample size 180). We simulate 1000 datasets for each model and calculate powers as described above.

**Table 4:** Ratio in F2 populations under different interaction models.

Model	A_B_	A_bb	aaB	aabb
No interaction	**9**	**3**	**3**	**1**
Dominant Epistasis	**12**		**3**	**1**
Recessive Epistasis	**9**	**3**	**4**	
Duplicate with cumulative effect	**6**	**6**		**1**
Duplicate Dominant	**15**			**1**
Duplicate Recessive	**9**	**7**		
Dominant & Recessive Interaction	**13**		**3**	

### Real Data

#### Age-related Macular Degeneration (AMD) data

The AMD dataset analyzed in this paper was published by Klein *et al* [26]. This dataset contains 103,611 SNPs (after primer QC) genotyped for 96 affected individuals and 50 controls. We removed SNPs containing more than four missing genotypes. After filtration, 96,607 SNPs remained. Then we applied *AprioriGWAS* with the default parameter setting on further quality-controlled data.

#### WTCCC Bipolar Disorder data

Bipolar disorder data used is available from WTCCC [27]. We take 1868 bipolar disorders versus 2938 controls genotyped on 393,271 SNPs for our genotype pattern search.

### Method Evaluation

#### Coverage of differential patterns comparison

To quantitatively estimate how many genuine differential patterns could be detected by *AprioriGWAS*, we compared significant differential patterns (pattern length = 2, significance level, *p*<0.0001) detected by the default setting of *AprioriGWAS* with an exhaustive search in 3200 simulated datasets. Coverage is defined as the percent of differential patterns detected in each simulated dataset.

#### Power comparison with logistic regression

To assess the power of different methods, we simulated 1000 cases and 1000 controls genotyped at 1,000 variants with a single pair of causative interacting loci. For each model and combination of parameters, we simulated 200 datasets. The power for each model and parameters is thus defined as the number of datasets from which we find the two simulated interaction variants divided by the total number of simulated datasets (here 200). To make a fair comparison of power, we control family-wise type I error by conducting permutation for both methods. Controlling the FDR (False Discovery Rate) of *AprioriGWAS* is done by conditional permutation, as stated above.

## Results

### Simulation Shows FDR Is Well Controlled by Conditional Permutation

We simulated data by two-locus interaction models proposed by Marchini *et al* [11] (**Methods**), in which three types of interactions are generated. We then applied regular permutation and conditional permutation to control family-wise type I error.

**Figure 1:**
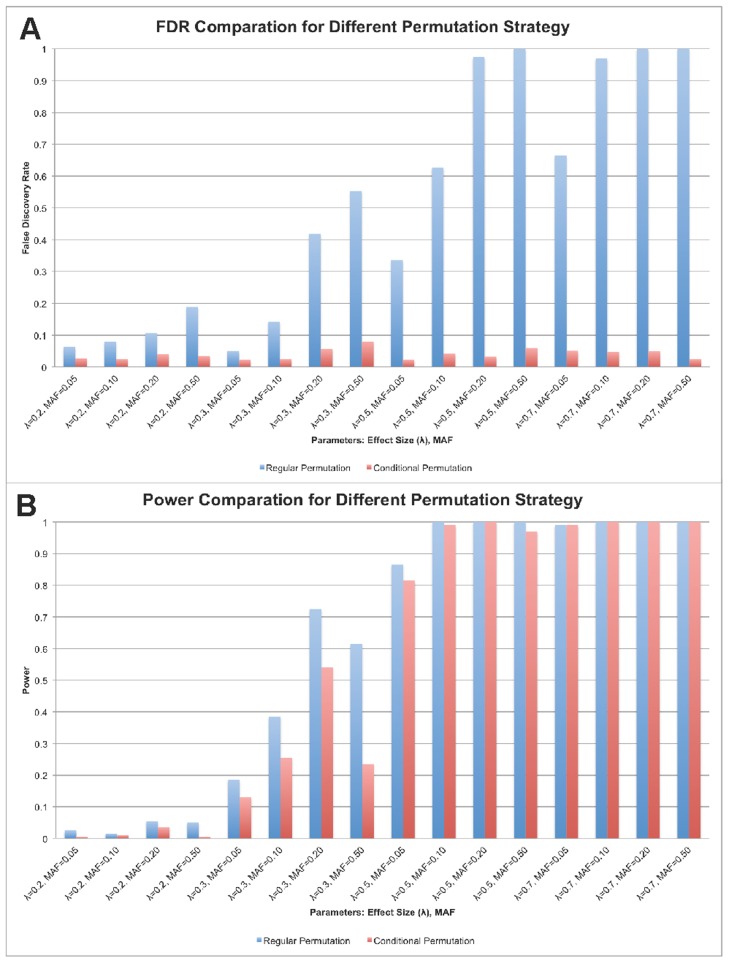
a. FDR comparison of regular permutation and conditional permutation; b. Power comparison of regular permutation and conditional permutation. FDR and power comparisons for regular permutation and conditional permutation (example results from epistasis Model 2). Y-axis is the power or FDR; X-axis shows combination of effect size (λ) and minor allele frequency (MAF) in simulation. (**a**): FDR comparison of controlling family-wise type I error ( = 0.05) by regular permutation and conditional permutation. (**b**): Power comparison of controlling family-wise type I error ( = 0.05) by regular permutation and conditional permutation.

The performances of regular permutation and conditional permutation test (**Methods**) are demonstrated in **Figure 1A and 1B**. We compared both power and FDR, using regular permutation and conditional permutations to adjust thresholds for type I error. Family-wise type I error was set to 0.05 for both methods. It is evident that the FDR was significantly reduced by the conditional permutation test, although some power is sacrificed compared with regular permutation.

**Figure 2:**
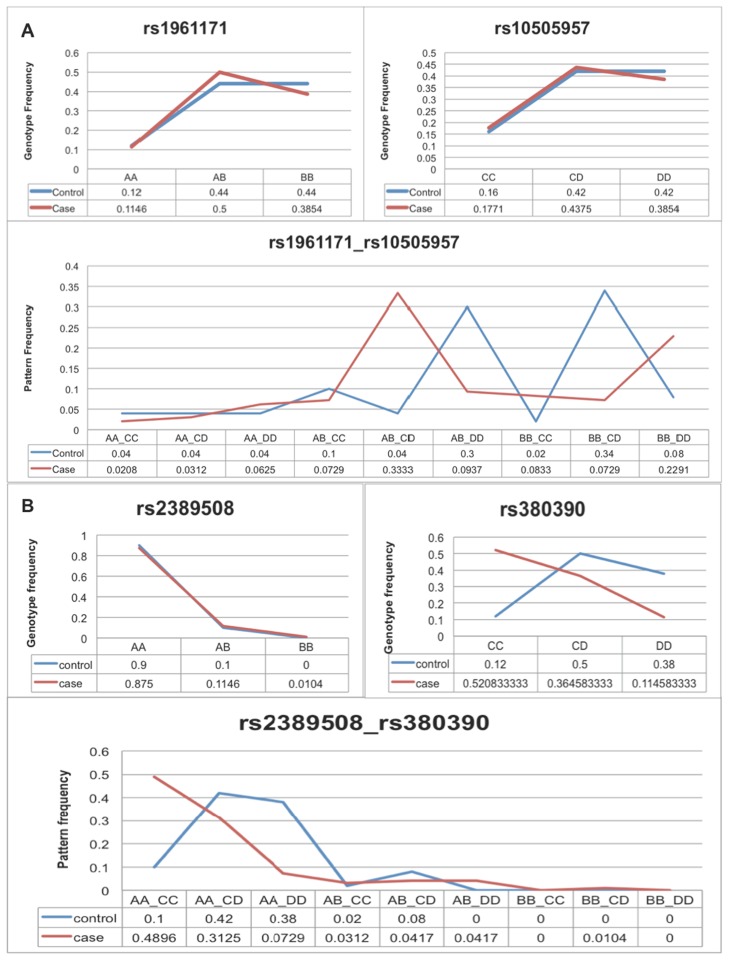
a. Patterns formed by variants without marginal effect; b. Patterns formed by variants with marginal effect. Evident examples justify the necessity of applying conditional permutation to control marginal effect from single variants. (**a**) The patterns formed by two neutral variants are more likely to be interacting, contrasting to (**b**) the low P-value of the contingency table is more likely due to the variants with strong marginal effect.

To demonstrate that the nominal p-value of a contingency table for multi-variants could be in large part caused by individual variants with strong marginal effect, we took a real example from analyzed AMD data. **Figure 2A** shows two variants, each with no marginal effect, but in combination with strong marginal effect. **Figure 2B** shows two variants, one has strong marginal effect, and the other does not show any marginal effect. Although the nominal p-value of the contingency table is more significant than the pair of variants in **Figure 2A**, one can deduce that the low p-value from **Figure 2B** is in large part caused by the variants with strong marginal effect; in **Figure 2A**, on the other hand, there must be some interaction effect.

### Coverage Comparison between Exhaustive Search and *AprioriGWAS*

As mentioned, *AprioriGWAS* manages to dramatically speed up the search process by dropping the candidate genotype patterns unlikely to grow to differential pattern. Since it is based on prediction at an early stage in the search, it still theoretically runs the risk of mistakenly dropping sensible patterns. Here we quantitatively tested the percentage of mistakenly dropped differential patterns by comparing *AprioriGWAS* and exhaustive search (**Method**).

**Figure 3:**
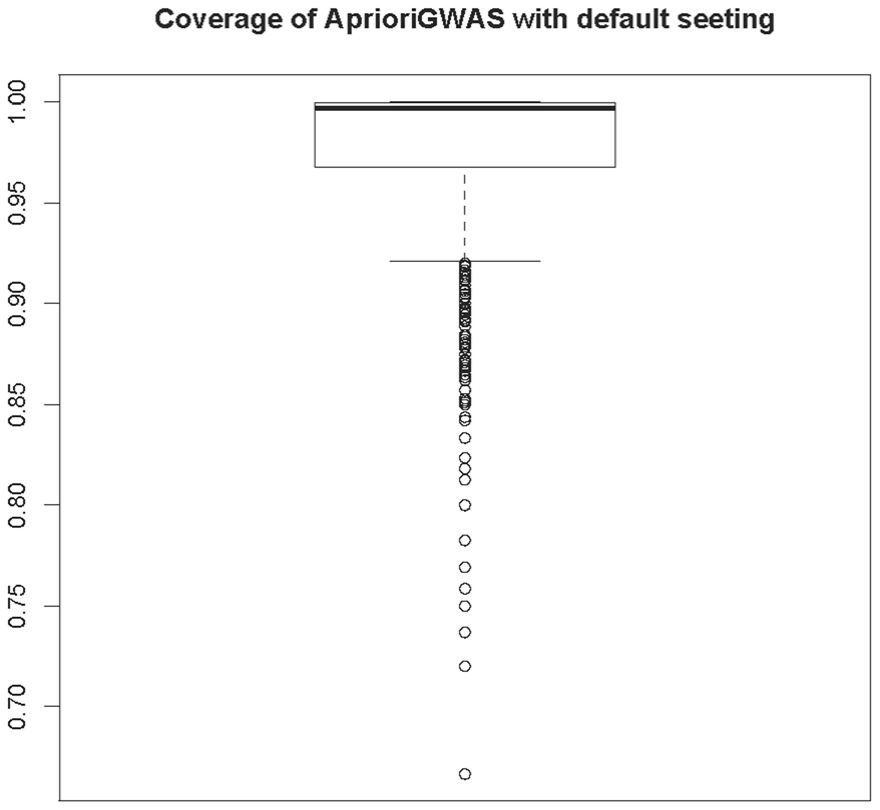
Coverage of finding differential genotype patterns by *AprioriGWAS*. Coverage comparison of *AprioriGWAS* with default setting and exhaustive search. On average 97 percent of differential patterns can be detected by *AprioriGWAS* with the default parameters setting.

**Figure 3** shows the comparison between searching for combinations of variants (with default parameters in *AprioriGWAS*) and exhaustive search. We found that 97% of all differential genotype patterns found by exhaustive search were covered by the results from *AprioriGWAS*. With such high coverage, the chance of losing possible interaction variants is minimized. There are a few points below 85%, reflecting that there is variation of power to cover all potential combinations. It is true that the overall coverage is subject to lots of parameters, like sample size and allele frequency. To minimize this variation, larger sample size is always desirable.

### Power Comparison between *AprioriGWAS*, Single Variant Test, and Logistic Regression (i.e., *epistasis* Function in PLINK) Using Theoretical Model

**Figure 4:**
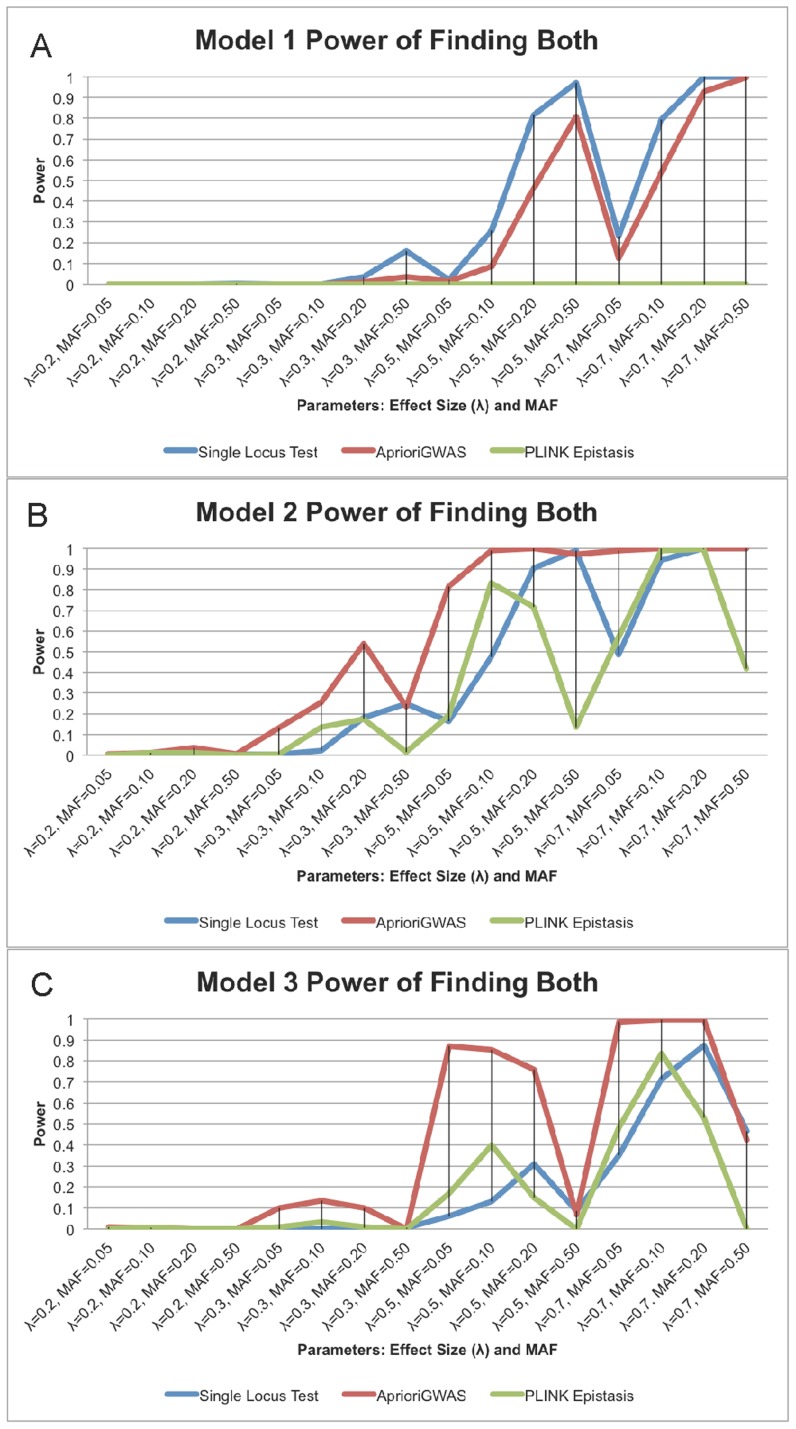
a. Power of finding both interacting variants for model 1; b. Power of finding both interacting variants for model 2; c. Power of finding both interacting variants for model 3. Power of finding both interacting variants for model 1, 2, and 3 (depicted in **a**, **b**, and **c** respectively). *AprioriGWAS* has much better power for Models 2 and 3, which do not show explicit marginal effect. The X-axis is the same as **Figure 1**.

We compared the ability of *AprioriGWAS* to find interacting variants with traditional single locus genotypic test and exhaustive search in PLINK [28] (*epistasis* function). The *epistasis* function in PLINK for case control data is basically stepwise logistic regression. We chose to use the all combinations option. The power comparison is based on two levels: finding at least one casual variant, or finding both interacting variants (**Figure 4**).

**Figure 5:**
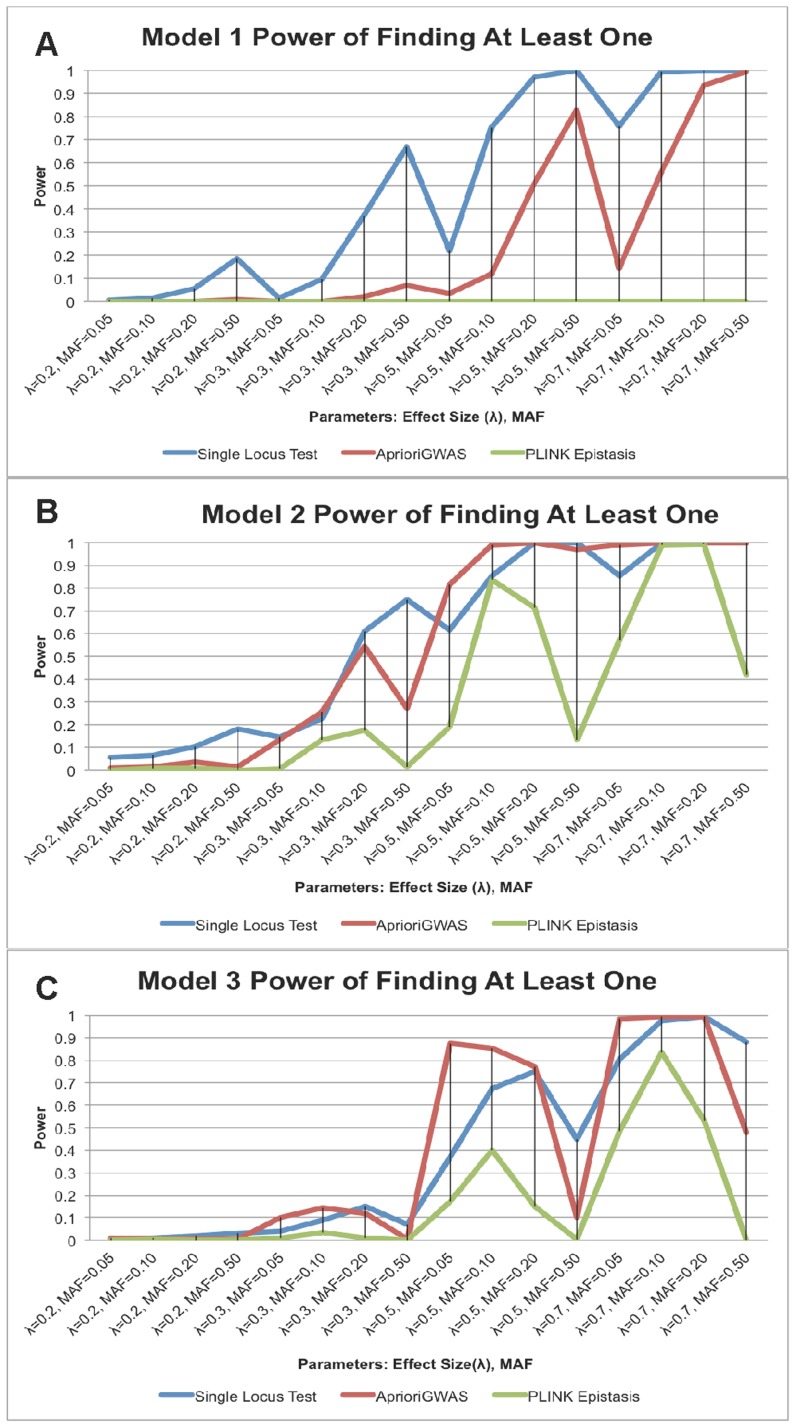
a. Power of finding at least one causal variant for model 1; b. Power of finding at least one causal variant for model 2; c. Power of finding at least one causal variant for model 3. Power of finding at least one casual variant for model 1, 2, and 3 (depicted in **a**, **b**, and **c** respectively). The single locus test has the highest power for Model 1, which has explicit marginal effect for both interacting variants; *AprioriGWAS* has better power for the threshold model, Model 3. The X-axis is the same as **Figure 1**.

For Level 1, detecting at least one causal variant, we found that the traditional single variant test had the highest power in Model 1, which has explicit marginal effects for both causal variants. *AprioriGWAS* performed similarly with the single loci test in Model 2, and had better power in Model 3 (**Figure 5**). This is natural, since Model 2 and 3, which contain no explicit marginal effects, are expected to be harder to detect without an interaction-based searching strategy.

For Level 2, detecting both interacting variants, it is evident that *AprioriGWAS* had the highest power in most cases of Model 2 and 3 (**Figure 4**). On the other hand, the performance of the epistasis function in PLINK, which exhaustively searches all combinations, was not as good in all cases. This is because: (1) stepwise logistic regression does not capture the interactions well, since the effects of the terms are added in a linear manner, whereas *AprioriGWAS* explicitly addresses detailed patterns; (2) in stepwise logistic regression the genuine interactions are buried by the noise of a too large number of combinations, whereas with the conditional permutation test used in *AprioriGWAS*, genuine interactions are able to stand out.

When comparing corresponding panels in **Figure 4** and **Figure 5**, it is observed that for the single variant test the power of finding both interacting variants (i.e., Level 2) dropped significantly compared with the power of finding at least one causal variant (i.e., Level 1). By contrast, interaction based methods, i.e., both *AprioriGWAS* and PLINK epitasis, maintained similar power for both levels. This was not unexpected since the interaction-based strategies should be better able to find an epistasis effect.

We also simulated data that have more SNPs (1,000,000) and find that the relative power between three methods and interaction models remain similar although the absolute powers are all decreased. (**Figure S1**)

### Power Comparison between *AprioriGWAS* and Single Variant Test Using Real Genotype and Studied Genetic Model

**Figure 6:**
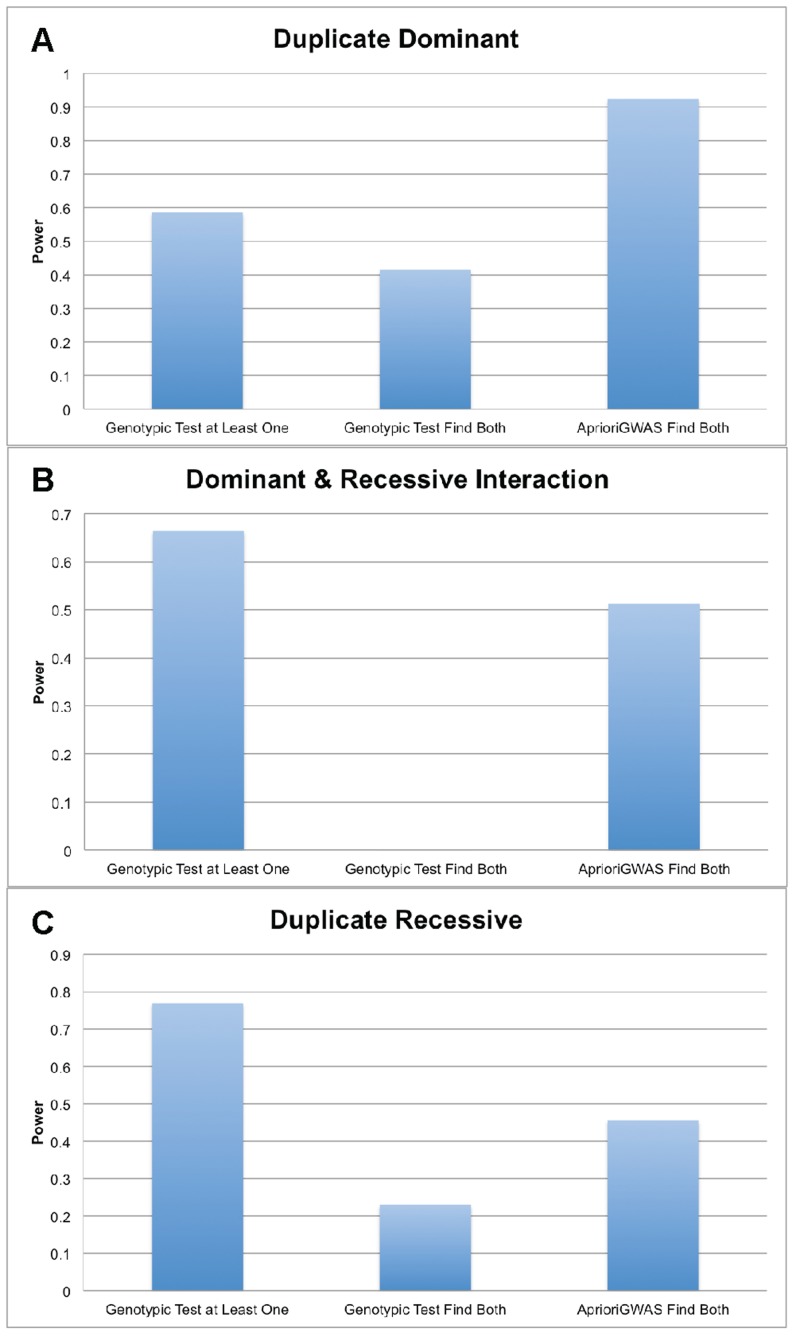
Power comparison using real genotype and known genetic models. a. Duplicate Dominat; b. Dominant & Recessive Interaction; c. Duplicate Recessive. Y-axis is power, X-axis denotes different methods.

**Figure 6** shows the power of *AprioriGWAS* and single variant test on three classical genetic models studied in model organisms. There are three powers for each genetic models: power for detecting at least one gene using single variant test, power for detecting both genes using single variant test, and power for detecting both genes using *AprioriGWAS*. Since PLINK is not scalable for such a dataset, we have not achieved power estimates for logistic regression. For the model “*Duplicated Dominant*”, *AprioriGWAS* outperforms single marker test for detecting single gene or both genes, whereas for models “*Duplicated Recessive*” and “*Dominant & Recessive Interaction*”, *AprioriGWAS* is more powerful for detecting both genes, but not for detecting single genes. It is notable that the power of detecting both genes in the model “*Dominant & Recessive Interaction*”, in which epistasis is functioning; single variant test has almost zero power (0.1%) while *AprioriGWAS* has around 50% power.

### CPU Time and Memory Usage

**Figure 7:**
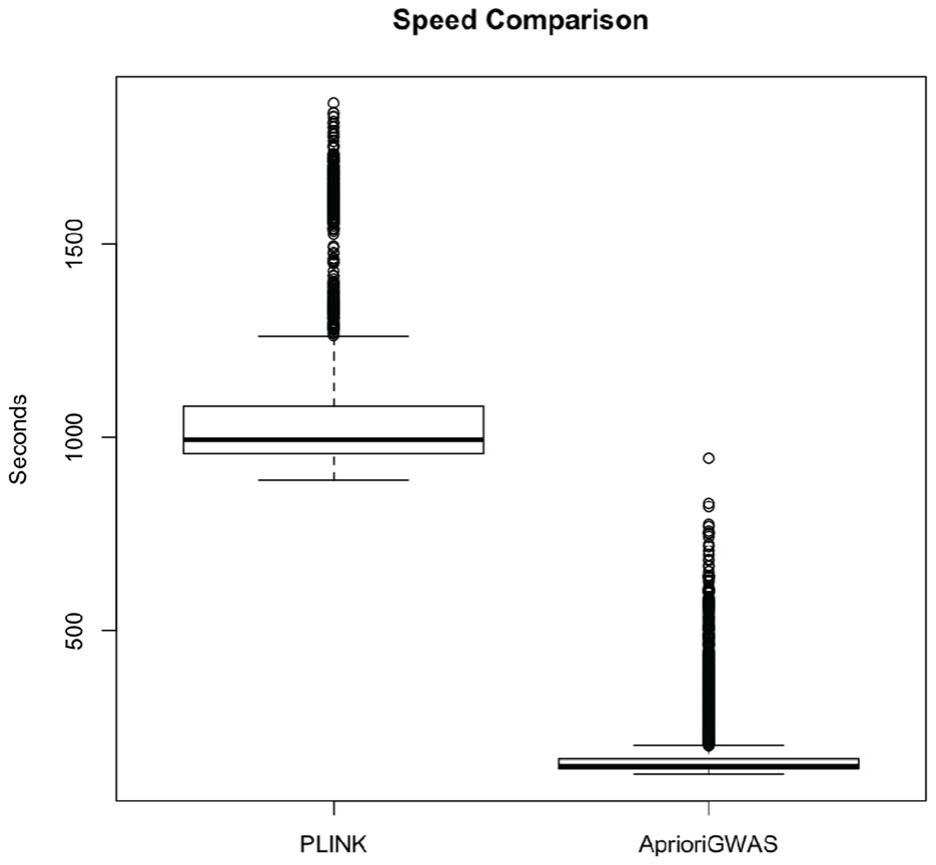
Speed comparing with Epistasis function in PLINK. CPU time compared with the epistasis function in PLINK. This comparison is based on 3200 simulated datasets, each with 1000 case, 1000 control and 1000 variants. *AprioriGWAS* with default parameters setting is a magnitude faster than PLINK.

We compared the speed of our method with the *epistasis* function in PLINK. **Figure 7** shows that the default threshold setting in *AprioriGWAS* was approximately a magnitude faster. Although retaining candidate genotype patterns in memory can help speed up the algorithm, its affordability is subject to the particular computational resources.

We took the strategy of writing candidate patterns on hard disk for each round of pattern extension. The genotype data used to be relatively small comparing with the patterns however is getting larger and larger empowered by new sequencing platforms. To solve this problem, we implemented *AprioriGWAS* using HDF5-based data format [29] which stores genotype data on disk and accesses them as though stored in main memory. Therefore, the memory usage is scalable to whatever size of potential dataset and the speed is not scarified. (See more on computational and memory complexity in section **Discussion**.)

### Applying *AprioriGWAS* to AMD Data

We applied *AprioriGWAS* on published AMD data [26]. We identified 168 significant pairs of variants (family-wise type I error = 0.01), presented in **Table S1**. By checking published functional literals and gene annotations, as well as GO enrichment of the genotype patterns, we learned that the findings are well validated by existing functional studies and clinical applications.

#### 1) Genes interacting with Complement Factor H

We found that ANGPT1, BBS9, PP7, MED27, CHRM2 interact with a well-known AMD gene, Complement Factor H (CFH). The most exciting finding here is ANGPT1, a protein with important roles in vascular development and angiogenesis. In fact, drugs for anti-angiogenic activity have been approved by the FDA in the United States for the treatment of cancer and AMD [30]. BBS9, MPP7, MED27, these three genes found here to interact with CFH have also been reported to be important for retinal disease [31]–[42].

#### 2) Verification from GO term enrichment

We performed GO term enrichment analysis for significant pairs of genes with an online tool developed by Roth lab (http://llama.mshri.on.ca/funcassociate/). **Table 5** lists enriched GO terms and their corresponding significant levels. Many studies from the fields of structural biology, translational biology, and immunology demonstrate that factor H recognizes and binds to self-surfaces via sialic acid and glycosaminoglycan (GAG) chains of proteoglycans, whereupon its complement-regulating properties were enhanced. The interaction between glycosaminoglycans (GAGs) and CFH plays an important role in the disease pathology of age-related macular degeneration [43]–[52].

**Table 5:** GO term enrichment analysis for AMD results.

N	X	LOD	P	P_adj	attrib ID	attrib name
**4**	22	1.669629349	5.303E-06	0.004	GO:0006024	glycosaminoglycan biosynthetic process
**4**	24	1.624997259	7.641E-06	0.005	GO:0006023	aminoglycan biosynthetic process
**4**	29	1.53008602	1.673E-05	0.011	GO:0030203	glycosaminoglycan metabolic process
**4**	33	1.466704223	2.835E-05	0.019	GO:0006022	aminoglycan metabolic process

#### 3) Replicated interactions found by other method on the same AMD data

The AMD data has also been analyzed by many other methods aiming to search gene-gene interaction. For example, Bayesian model based method, BEAM [53] and epiMODE [8] and forest-based approaches [54] and [55]. In their forest-based approaches on the same dataset we are using, Chen *et al* [54] and Wang *et al* [55] found a haplotype in gene BBS9 interacting with a haplotype in the CFH gene. Our results confirm theirs.

### Applying *AprioriGWAS* to WTCCC Bipolar Disorder Data

Besides AMD data that were extensively analyzed by the community interested in gene-gene interactions, we also applied *AprioriGWAS* on Bipolar Disorder data from WTCCC [27] to further test whether it is scalable for larger dataset. The whole task was distributed onto 1,000 CPUs in a cluster and the average execution time for a single job is 56.8 hours. Only 4 Gb memories were employed during the computation, evidencing the great performance of HDF5-based implementations.

#### 1) Variants without marginal effect show significant interactions

Based on 1000 conditional permutations, we identified 200 significant pairs of variants (family-wise type I error = 0.001), presented in **Table S2**. The observed number of interactions is inflated due to LD. Majority of interacting variants doesn't show marginal effect in single variants test. One important aspect of *AprioriGWAS* is that people can always check genotype patterns that drive the contingency table of variants combination to be significant.

#### 2) Evidence from GO term enrichment analysis

Go terms “synaptic membrane” (GO:0097060), “synaptic transmission” (GO:0007268), “transmission of nerve impulse” (GO:0019226) and “multicellular organismal signaling” (GO:0035637) are barely significantly enriched in found SNPs pairs (**Table 6**).

**Table 6:** GO term enrichment analysis for Bipolar Disorder from WTCCC.

N	X	LOD	P	P_adj	attrib ID	attrib name
**7**	215	0.937543575	4.83E-05	0.072	GO:0097060	synaptic membrane
**12**	664	0.684053863	3.53E-05	0.061	GO:0007268	synaptic transmission
**13**	754	0.666216413	2.63E-05	0.05	GO:0019226	transmission of nerve impulse
**13**	781	0.649980196	3.79E-05	0.07	GO:0035637	multicellular organismal signaling

#### 3) Evidence from public database and literatures

We found multiple-SNP genotype patterns inside individual genes (8 out of 18 genes in **Table S2** are related with mental disorder). Good examples are GABRB2 and GRIA1 (α-amino-3-hydroxy-5-methyl-4-isoxazole proprionic acid (AMPA) subunit 1 receptor gene). AMPARs are found in many parts of the brain and are the most commonly found receptor in the nervous system. The GABRB2 mediates the fastest inhibitory synaptic transmission in the central nervous system. Multiple evidences showed that GRIA1 and GABRB2 are relevant to Bipolar Disorder and Schizophrenia [56]–[62]. These genes however haven't been found in original analysis of WTCCC bipolar disorder using single marker tests. We also identified interactions across genes or chromosomes. Focus only on multiple hit of interact regions, GRIK3 from chromosome 1 interacts with a region in chromosome 3; SULT4A1 from chromosome 22 interacts with a region on chromosome 12; LRFN2 from chromosome 6 interacts with SORBS1 from chromosome 10; Based on queries from GeneCard database (http://www.genecards.org), we found that diseases associated with GRIK3, SULT4A1 and LRFN2 are schizophrenia, schizotypal personality disorder and neuronitis respectively, and SORBS1 is associated with insulin resistance.

#### 4) Result by other gene-gene interaction method

Most interaction studies for bipolar disorder focuses on gene-environment interaction rather than gene-gene interaction. There is a literature focusing on gene-gene interaction, Oh *et al* [63], that also identified that GABRB2 plays important role in Bipolar Disorder.

## Discussion

We have introduced *AprioriGWAS*, patterned after the *Apriori* algorithm in the bioinformatics field of frequent itemset mining (FIM), as a tool for detecting main and interaction effects of genetic variants in case-control association studies. One of its outstanding properties is that it can find variants whose disease association lives solely from their interaction without having (appreciable) main effects. We applied our approach to a published dataset on AMD and documented that *AprioriGWAS* furnishes sensible results. In fact, it found an AMD-associated variant (ANGPT1) not previously reported to be associated with AMD. We also identified interesting genes from WTCCC bipolar disorder data. One good point is that GO term enrichment analyses of all the genes identified, always show sensible terms for relevant disease. Our description of these findings is primarily intended to show the efficacy of our approach rather than to provide research findings about AMD and bipolar disorder.

### False Discovery and Replication in Other Dataset

Regardless the goal being interaction or single gene, statistical tests all suffer from the problem of false positives. Since the numbers of variants (and their combinations) are usually a few magnitudes larger than the sample size for most association studies, it will be common to see false positives. The current practice in the community is that researchers who would like to claim association or carry out experimental validations usually have to check whether the results are replicable in other independent dataset(s) Researchers who use *AprioriGWAS* can also use this to filter results before doing experimental validations. As an example, we use another independent dataset for AMD study [64] to check whether the results are replicable. Among the five interactions with CFH reported in this paper, we found that BBS9/CFH and CHRM2/CFH are replicated in the other dataset. However, we understand that these two datasets are very different: one is wet AMD and the other is dry AMD. One of them is more prevalent in Asia than the other. Therefore, our further analysis of data in [64] may not serve as perfect replication of the findings presented, although it suggests that BBS9 and CHRM2 may be of higher priority for further experimental validations.

### Other Multiple Variants Analysis Methods

The most commonly used multiple variants analysis is stepwise regression, in which variants are added to the regression equation one after another by some suitable criteria. But statistical analysis shows that the usual stepwise model selection methods are path dependent and therefore suboptimal [65]. Besides regression, some methods are based on discrete mathematics, like the Combinatorial Partition Method (CPM) [66] and its refined version, the Restricted Partition Method (RPM) [67]. However, RPM still requires a daunting number of tests when the number of variants is high. This is because its insight into reducing tests lies in its practice to combine close phenotypes, which consequently does not entirely solve the problem of too many combinations of genotypes. Another well-known method of counting potential combinations is multifactor dimensionality reduction (MDR). It collapses cells in a contingency table into two groups and conducts a test on them. Essentially however it reduces the dimensionality of testing, rather than reducing the dimensionality of the process of counting genotype patterns. Therefore, when the number of variants is large, it still suffers from the “curse of dimensionality” [17]. Bayesian methods leveraging MCMC, e.g, BEAM [53] or epiMODE [8], should theoretically suffer less from computational limitations, but they do not directly test detailed combinations of genotype patterns and thereby sacrifice the advantages of fine scale learning of gene-gene interactions. Another branch of frequently used methods is two-stage analysis [68], by which the investigator can utilize relatively “simple” or computationally efficient tests to choose qualified variants in the first stage analysis. Then, taking advantage of the relatively small number of variants, the investigator can adopt some advanced but computationally heavy test to identify interacting genes. However, due to a lack of strong prior knowledge, the true signals might have been removed from the first stage if the procedure was not well designed. As an example, interacting variants with no marginal effect may be filtered out if one uses tests based on marginal effects of single variants in the first stage. Nevertheless, with good design, this approach is still very promising and can be combined with all the approaches reviewed above; and it can naturally also be combined with the method proposed in this work.

### Computation Time and Algorithm Complexities

Computation time and spatial complexities of the tool may be interesting to the reader. The number of transactions for original *Apriori* corresponds to sample size in GWAS; the number of items is equivalent to the number of variants and the itemsets. In contrast to supermarket data, GWAS data have a limited number of “transactions”, but a large number of “items” in two datasets, cases and controls. Both conditions make the problem more difficult. The time spent reading the data in each round of pattern growth is constant. In addition, the computational resources cost depends on how many combinations of genetic variants will be generated and tested. The more combinations are tested, the less likely it is that genuine patterns are missed, though of course more resources will be used. In *AprioriGWAS*, there are several parameters for the user to specify according to their computer resources and understanding of the disease model. The threshold for the proportion test and minimal support of concerned itemsets are parameters that affect candidate search space, algorithm speed, and power of detecting all distinct genotype patterns. When these parameters are set to zero, *AprioriGWAS* will exhaustively search all possible combinations. (Please refer to our Manual of *AprioriGWAS* for the tradeoffs and discussions on setting these parameters according to computational resources.)

Those familiar with *Apriori* may suggest that, given *Apriori*'s ability to also mine association rules, one could also treat the case control label as items and directly adopt *Apriori* for case/control data. The result will then be a subset of variants that can imply the case/control labels. But searching frequent itemsets and then mining the association between genotype pattern and disease status is inefficient, since frequent genotype patterns are not necessarily associated with phenotype; on the other hand, genotype patterns strongly associated with phenotype may not necessarily be in high frequency, and such an association could be distributed in different patterns than the same variants combinations.

### Conditional Permutation versus Regular Permutation on Controlling Family-Wise Type I Error

Instead of the conditional permutation proposed here, one could also consider Bonferroni correction. For *n* variants with search length of *m*, the total number of combinations is huge. Given the natural correlation of the combinations, it is clearly far more stringent than necessary. However, only correcting on the number of differential pattern tested produces a bias in the other direction, since the nominal value of the significance level of the chi-square test for the 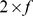 contingency table will be inflated by the selection procedure [69]. It is therefore always preferable to use a permutation test for the whole procedure. With regular permutation, one permutes the Case/Control label and then performs the whole test process. The smallest P-value of each permutation are ranked, allowing one to get the distribution of test statistics under “Null” from the permuted dataset. With regular permutation, no variant should have marginal effect, and the p-value of the contingency table for the combination of variants is under the null hypothesis of no variants having marginal effect.

However, regular permutation suffers from an inflated significance level for contingency tables containing variants with marginal effects. This is due to the fact that when a contingency table is composed of at least one variant with strong marginal effect, the p-value for that contingency table becomes extremely small compared with regular permutation results. The FDR is therefore very high, even close to 1.

To solve the problem of an inflated significance level by a contingency table composed of at least one variant, *v*, with strong marginal effect, we developed a conditional permutation procedure (**Methods**), which helps get the null distribution of the p-value of a contingency table composed of the variant and other variants. Simulation results show that, when we control the family-wise type I error by conditional permutation, we also keep FDR well controlled. Compared with INTERSNP [13], which lists only the top 50 variant combinations including the variant with marginal effect, conditional permutation in *AprioriGWAS* keeps FDR well controlled in a systematic way.

### Linkage Disequilibrium (LD)

Another concern might be whether these differential genotype patterns are artifacts caused by linkage disequilibrium (LD). We believe this is not the case, since the LD should impact both cases and controls, and therefore the pattern created by LD will not be differential unless the LD structure is significantly different in cases and controls for particular genetic variants. If that is the case, then there must be some reason of selection to explain the deviation in the genotype pattern, and it is difficult to judge whether this is an artifact or something of interest. In addition, our conditional permutation also breaks LD between interacting variants.

### Rare and Low-Frequency Variants

Low-frequency or rare variation might impact the performance of the method, even when explicitly only testing for interactions among common variants. What matters is the extent of LD between causal rare variants and testing common variants. We haven't addressed this problem in the current method. It would be interesting to extend *AprioriGWAS* toward that direction. There may be non-trivial statistical challenges since the low-frequency or rare variants are usually less shared by the individuals therefore their combinations that form genotype patterns will be even less shared by individuals. For a given set of variants, we will have many patterns with little supports.

## Supporting Information

Figure S1 **Power comparison using 1,000,000 genetic variants. a. Power of finding both interacting variants for model 1; b. Power of finding both interacting variants for model 2; c. Power of finding both interacting variants for model 3.** Power of finding both interacting variants for model 1, 2, and 3 (depicted in **a**, **b**, and **c** respectively). *AprioriGWAS* has much better power for Models 2 and 3, which do not show explicit marginal effect. The X-axis is the same as **Figure 1** & **Figure 5**.
(TIFF)

Table S1 **Results of Age related Macular Degeneration (AMD).** 168 pairs of variants show significant genotype pattern difference between case and control samples.
(PDF)

Table S2 **Results of Bipolar Disorder from WTCCC.** 200 pairs of variants show significant genotype pattern difference between case and control samples.
(PDF)
