# Supplementary figures and images for: AprioriGWAS, a New Pattern Mining Strategy for Detecting Genetic Variants Associated with Disease through Interaction Effects
Source: PLoS Comput Biol. 2014 Jun 5;10(6):e1003627. doi: 10.1371/journal.pcbi.1003627 (PMC4046917; doi:10.1371/journal.pcbi.1003627)

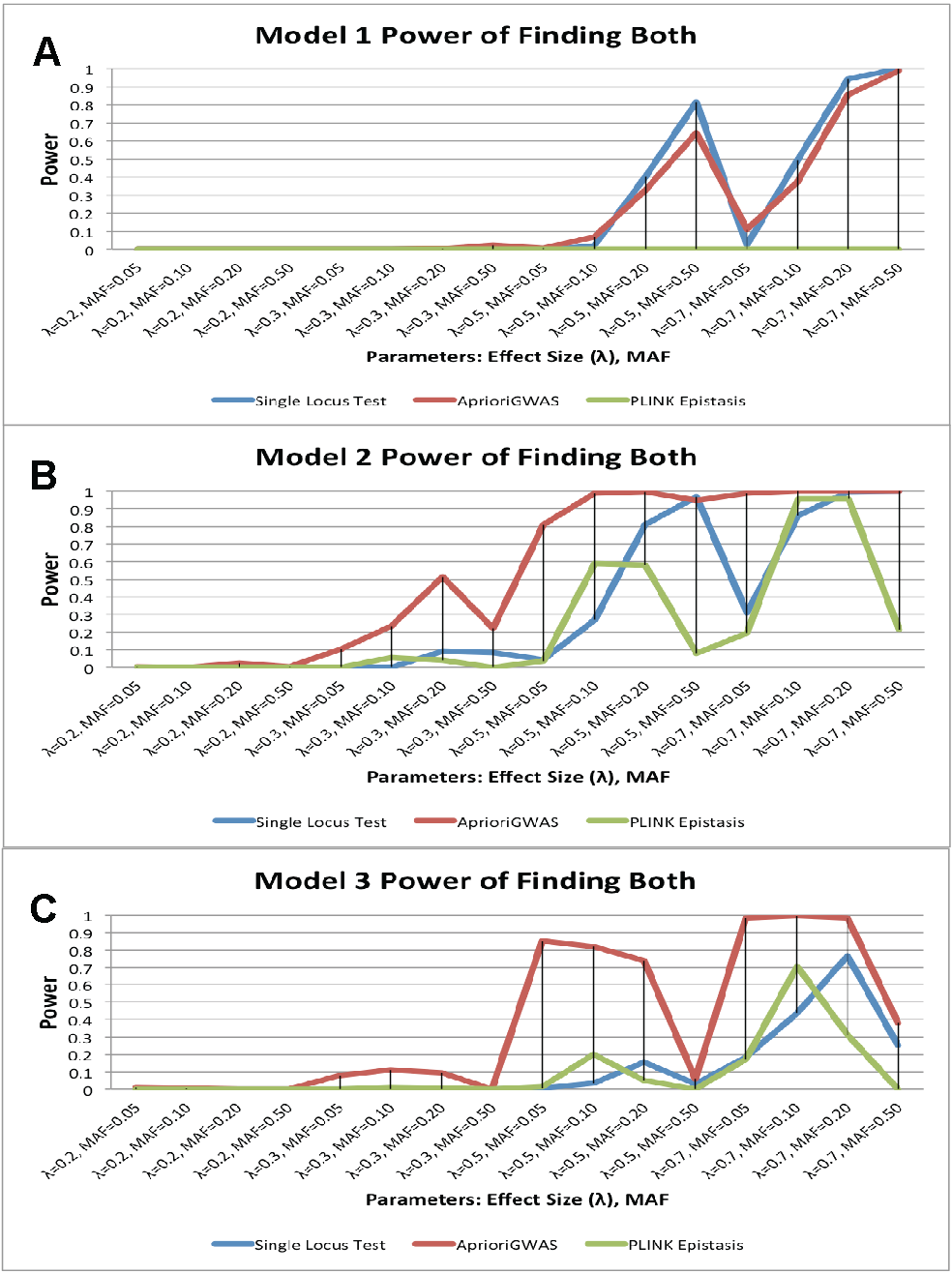

Supplement: Figure S1 — Power comparison using 1,000,000 genetic variants. a. Power of finding both interacting variants for model 1; b. Power of finding both interacting variants for model 2; c. Power of finding both interacting variants for model 3. Power of finding both interacting variants for model 1, 2, and 3 (depicted in a, b, and c respectively). AprioriGWAS has much better power for Models 2 and 3, which do not show explicit marginal effect. The X-axis is the same as Figure 1 & Figure 5 . (TIFF) [file pcbi.1003627.s001.tif]
