# Supplementary material for: AprioriGWAS, a New Pattern Mining Strategy for Detecting Genetic Variants Associated with Disease through Interaction Effects
Source: PLoS Comput Biol. 2014 Jun 5;10(6):e1003627. doi: 10.1371/journal.pcbi.1003627 (PMC4046917; doi:10.1371/journal.pcbi.1003627)
Supplement: Table S1 — Results of Age related Macular Degeneration (AMD). 168 pairs of variants show significant genotype pattern difference between case and control samples. (PDF) [file pcbi.1003627.s002.pdf]

Table S1 | Results of Age related Macular Degeneration (AMD).

SNP_1	Chr.	Loc.	Gene	SNP_2	Chr.	Loc.	Gene	Nominal_p_value
rs10231824	chr7	131922332	PLXNA4	rs571361	chr1	82126526	.	4.42E-08
rs339884	chr18	22255158	.	rs195937	chr4	163580271	.	8.88E-08
rs2015222	chr16	52024688	.	rs10506877	chr12	83045954	.	2.74E-07
rs4784047	chr16	52016720	.	rs10506877	chr12	83045954	.	2.74E-07
rs10518739	chr15	68014971	MAP2K5	rs931210	chr5	113092959	.	3.90E-07
rs1072173	chr14	94738364	PPP4R4	rs10506877	chr12	83045954	.	2.74E-07
rs1844510	chr14	63217488	KCNH5	rs6421760	chr1	96296450	.	2.36E-07
rs7154189	chr14	63083581	.	rs10516495	chr4	103900090	SLC9B1	2.62E-07
rs7154189	chr14	63083581	.	rs756308	chr10	19520364	.	2.62E-07
rs7154360	chr14	63083549	.	rs10516495	chr4	103900090	SLC9B1	2.62E-07
rs7154360	chr14	63083549	.	rs756308	chr10	19520364	.	2.62E-07
rs10483466	chr14	36917076	.	rs10494671	chr1	192324175	RGS21	2.91E-07
rs12323203	chr13	82087781	.	rs951300	chr3	149455651	.	3.35E-07
rs9318185	chr13	74101515	.	rs10485181	chr6	116674838	DSE	3.06E-07
rs7273825	chr20	38215274	.	rs9287251	chr1	240954521	RGS7	3.93E-07
rs9304420	chr18	49819796	.	rs1165017	chr9	138602228	KCNT1	2.05E-07
rs2278031	chr16	71768500	AP1G1	rs446815	chr11	115819664	.	3.88E-07
rs10507601	chr13	56149378	.	rs1436532	chr4	96797215	.	9.23E-08
rs1473216	chr12	27983910	.	rs717246	chr1	79329349	.	3.46E-07
rs10501935	chr11	99811973	CNTN5	rs1695943	chr1	55979499	.	2.54E-07
rs587334	chr11	90350730	.	rs10509236	chr10	67569589	.	2.48E-07
rs10500875	chr11	20343326	.	rs2132292	chr3	76294385	.	1.80E-07
rs6483628	chr11	20005278	NAV2	rs10504003	chr8	37128540	.	3.96E-07
rs7087186	chr10	60523725	BICC1	rs7074652	chr10	54770299	.	3.43E-07
rs756308	chr10	19520364	.	rs2011542	chr7	97290158	.	1.15E-08

rs10508580	chr10	19511432	.	rs2011542	chr7	97290158	.	3.55E-07
rs10508579	chr10	19509393	.	rs2011542	chr7	97290158	.	1.49E-07
rs9314750	chr9	88896956	ISCA1	rs195937	chr4	163580271	.	3.92E-07
rs10511550	chr9	10648555	.	rs951300	chr3	149455651	.	3.35E-07
rs285875	chr8	106055586	.	rs1454498	chr2	70149225	MXD1	3.35E-07
rs2011542	chr7	97290158	.	rs10516495	chr4	103900090	SLC9B1	1.15E-08
rs10259490	chr7	49616420	.	rs10517155	chr4	29203553	.	3.26E-07
rs2248634	chr7	21291878	.	rs10497709	chr2	191656929	.	1.10E-07
rs1557099	chr6	154293295	.	rs259065	chr5	79100717	.	2.62E-07
rs2346528	chr6	112881853	.	rs6701925	chr1	239811762	CHRM3	2.71E-07
rs2322840	chr6	81262941	.	rs10489479	chr1	176582976	PAPPA2	2.50E-07
rs1362932	chr6	46716079	ANKRD66	rs1964092	chr2	7153693	RNF144A	1.88E-07
rs9296510	chr6	46713805	.	rs1964092	chr2	7153693	RNF144A	2.51E-07
rs3797564	chr5	115239004	AP3S1	rs1964092	chr2	7153693	RNF144A	1.81E-07
rs305728	chr5	84634092	.	rs10497071	chr2	151608765	.	9.22E-08
rs149517	chr5	53370659	ARL15	rs10512934	chr5	7936287	.	3.01E-07
rs39883	chr5	53369015	ARL15	rs10512934	chr5	7936287	.	1.74E-07
rs2726526	chr4	106292121	PPA2	rs6756237	chr2	175019633	OLA1	3.70E-07
rs1866989	chr4	47101105	GABRB1	rs10492958	chr1	217124015	ESRRG	3.84E-07
rs1866989	chr4	47101105	GABRB1	rs10492960	chr1	217122670	ESRRG	3.84E-07
rs4856145	chr3	103055089	.	rs3828057	chr1	151780177	RORC	4.94E-08
rs7632290	chr3	29484510	RBMS3	rs10497881	chr2	205992964	PARD3B	2.05E-07
rs3771649	chr2	159473554	PKP4	rs1892145	chr1	192328455	RGS21	3.86E-07
rs10497071	chr2	151608765	.	rs1534185	chr2	124157232	.	2.10E-07
rs968051	chr2	104453225	.	rs7559494	chr2	82816618	.	1.91E-07
rs2836048	chr21	39284298	KCNJ6	rs2828516	chr21	25135471	.	5.11E-08
rs10521129	chr17	4008820	ZZEF1	rs3844556	chr10	102072092	PKD2L1	1.87E-08
rs10521127	chr17	3978268	ZZEF1	rs3844556	chr10	102072092	PKD2L1	2.55E-08

rs10519680	chr15	32030697	.	rs10503703	chr8	21215716	.	9.43E-08
rs912304	chr14	25476170	STXBP6	rs1039336	chr9	8376287	PTPRD	2.36E-07
rs854331	chr14	25328163	STXBP6	rs649031	chr13	51355243	DLEU7	1.85E-07
rs314395	chr13	89808242	.	rs224119	chr10	64443400	.	2.53E-07
rs2413151	chr22	33167869	SYN3	rs718685	chr2	218386084	DIRC3	1.63E-07
rs722557	chr21	39278677	KCNJ6	rs709274	chr2	240331971	.	2.38E-07
rs720202	chr16	83474471	CDH13	rs10508291	chr10	4810991	.	2.40E-07
rs2343866	chr12	26315082	.	rs10486157	chr7	7350683	.	1.50E-07
rs1060447	chr10	100218532	HPSE2	rs1397196	chr6	124340533	NKAIN2	1.39E-07
rs6494691	chr15	68009575	MAP2K5	rs6948174	chr7	69511919	AUTS2	5.72E-08
rs649031	chr13	51355243	DLEU7	rs1392296	chr3	46143930	.	1.85E-07
rs249847	chr12	98867716	.	rs1857393	chr9	126497262	DENND1A	2.26E-07
rs1565216	chr12	95735183	.	rs224119	chr10	64443400	.	2.53E-07
rs10506115	chr12	33845977	.	rs1408120	chr9	9756718	PTPRD	1.95E-07
rs10506115	chr12	33845977	.	rs9324943	chr5	143570841	KCTD16	1.95E-07
rs2298809	chr11	123554086	.	rs7688514	chr4	177684985	VEGFC	2.96E-08
rs1986404	chr11	58194614	.	rs853727	chr4	149835012	.	1.51E-07
rs1986404	chr11	58194614	.	rs7678724	chr4	149867965	.	1.51E-07
rs10509274	chr10	68469322	CTNNA3	rs2356376	chr10	16752483	RSU1	1.93E-07
rs718309	chr10	55781181	PCDH15	rs501138	chr10	29489709	.	1.55E-07
rs718309	chr10	55781181	PCDH15	rs4880042	chr9	36950301	PAX5	7.87E-08
rs1857393	chr9	126497262	DENND1A	rs1337620	chr9	1151269	.	2.26E-07
rs1857393	chr9	126497262	DENND1A	rs7570567	chr2	157418626	GPD2	2.26E-07
rs1857393	chr9	126497262	DENND1A	rs2300564	chr1	168391067	LOC100505918	2.26E-07
rs9298846	chr9	23226243	.	rs994542	chr6	63247098	.	5.22E-09
rs2581482	chr8	122801173	.	rs10495668	chr2	17908881	SMC6	8.65E-08
rs10504339	chr8	62836663	.	rs10496106	chr2	64346664	PELI1	1.57E-07
rs10504339	chr8	62836663	.	rs3801740	chr7	81781051	CACNA2D1	1.57E-07

rs10500148	chr7	144675704	.	rs10495668	chr2	17908881	SMC6	1.89E-07
rs725859	chr7	123424583	.	rs10486015	chr7	106221184	.	2.29E-07
rs6948174	chr7	69511919	AUTS2	rs1961273	chr2	137602926	.	5.72E-08
rs10486521	chr7	33238200	BBS9	rs248364	chr5	160686633	.	1.26E-07
rs10225559	chr7	28903231	.	rs10484566	chr6	32835258	.	1.74E-07
rs10486157	chr7	7350683	.	rs9288014	chr2	178950399	PDE11A	2.58E-07
rs2342665	chr4	156100421	.	rs10495668	chr2	17908881	SMC6	1.74E-07
rs2695214	chr4	102079736	PPP3CA	rs10511298	chr3	111267564	CD96	1.44E-07
rs5743072	chr2	190696924	PMS1	rs2999859	chr1	163166723	RGS5	1.44E-07
rs1852556	chr2	132095028	.	rs261796	chr1	241110180	RGS7	1.20E-07
rs555174	chr21	20923493	.	rs10510185	chr3	195923	.	2.20E-07
rs2899194	chr22	33167851	SYN3	rs718685	chr2	218386084	DIRC3	1.63E-07
rs10502291	chr18	928190	.	rs1510134	chr4	143901714	.	1.05E-07
rs1394572	chr16	78911336	WWOX	rs6598991	chr1	82985967	.	1.33E-07
rs4321143	chr15	96156368	.	rs962848	chr11	44204159	EXT2	1.33E-07
rs719737	chr13	110350349	.	rs10501439	chr11	79085846	TENM4	9.78E-08
rs9300408	chr13	98047677	.	rs7006908	chr8	67193868	.	5.94E-08
rs1972634	chr19	34352547	.	rs10512174	chr9	88886574	ISCA1	2.80E-08
rs7235818	chr18	58236573	.	rs725518	chr11	4128845	RRM1	6.53E-08
rs727454	chr18	54045119	.	rs7698633	chr4	84982151	.	5.29E-08
rs10513889	chr18	54011876	.	rs7698633	chr4	84982151	.	5.29E-08
rs2323284	chr17	14410166	.	rs7006908	chr8	67193868	.	4.48E-08
rs2474584	chr10	38416389	.	rs3773563	chr3	55059144	CACNA2D3	4.65E-08
rs176880	chr10	38401417	ZNF37A	rs3773563	chr3	55059144	CACNA2D3	4.65E-08
rs10492272	chr12	99265148	ANKS1B	rs4472954	chr11	80920926	.	1.20E-07
rs1472874	chr12	24575272	SOX5	rs7006908	chr8	67193868	.	9.77E-08
rs2513240	chr11	86794856	TMEM135	rs10512174	chr9	88886574	ISCA1	7.68E-08
rs10501439	chr11	79085846	TENM4	rs7870469	chr9	102859802	ERP44	4.02E-08

rs962848	chr11	44204159	EXT2		rs909786	chr6	16659095	ATXN1	7.32E-08
rs1467644	chr10	99961158	R3HCC1L		rs7006908	chr8	67193868	.	5.94E-08
rs1548255	chr10	38417193	.		rs3773563	chr3	55059144	CACNA2D3	4.65E-08
rs10508731	chr10	28442788	MPP7		rs359512	chr4	162881087	FSTL5	9.56E-08
rs10512174	chr9	88886574	ISCA1		rs10511902	chr9	32323271	.	7.44E-08
rs7006908	chr8	67193868	.		rs10490631	chr2	118678439	CCDC93	7.15E-08
rs7006908	chr8	67193868	.		rs961360	chr2	136393658	R3HDM1	8.29E-08
rs7006908	chr8	67193868	.		rs1461774	chr3	194439908	LOC100507391	6.18E-08
rs7006908	chr8	67193868	.		rs93059	chr4	103468518	NFKB1	4.97E-08
rs7006908	chr8	67193868	.		rs10254116	chr7	33237489	BBS9	8.33E-09
rs10488343	chr7	131574321	.		rs4920799	chr5	84606528	.	1.21E-08
rs10254116	chr7	33237489	BBS9		rs7765392	chr6	135005295	.	1.34E-07
rs10254116	chr7	33237489	BBS9		rs2182277	chr6	6958261	.	8.75E-08
rs10486459	chr7	25525767	.		rs1146382	chr1	85918101	DDAH1	5.31E-09
rs9296242	chr6	38210712	BTBD9		rs551591	chr1	53301876	.	1.16E-07
rs2987582	chr6	8688087	LOC100506207		rs551591	chr1	53301876	.	5.53E-08
rs9328470	chr6	8396748	.		rs551591	chr1	53301876	.	6.87E-08
rs1510134	chr4	143901714	.		rs1388612	chr3	62244444	PTPRG	5.90E-08
rs10495405	chr1	238287030	.		rs551591	chr1	53301876	.	1.34E-07
rs1340465	chr1	238286612	.		rs551591	chr1	53301876	.	1.34E-07
rs1538444	chr1	238282502	.		rs551591	chr1	53301876	.	1.34E-07
rs555174	chr21	20923493	.		rs7006908	chr8	67193868	.	3.10E-08
rs200642	chr20	51943032	TSHZ2		rs7176701	chr15	73989821	CD276	4.61E-08
rs6104678	chr20	10934207	.		rs4652920	chr1	37088494	.	1.14E-07
rs6104678	chr20	10934207	.		rs966978	chr3	87567430	.	3.31E-08
rs6104678	chr20	10934207	.		rs2945861	chr8	8283667	.	1.23E-07
rs6104678	chr20	10934207	.		rs1468884	chr18	54861038	.	2.29E-09
rs10507949	chr13	85856134	.		rs1731298	chr7	53026187	.	1.25E-08

rs10507949	chr13	85856134	.	rs123491	chr4	23425990	MIR548AJ2	3.96E-09
rs10507949	chr13	85856134	.	rs10511467	chr9	7373051	.	2.36E-08
rs10502376	chr18	8589765	.	rs10511467	chr9	7373051	.	1.31E-08
rs1394608	chr5	155783294	SGCD	rs6847164	chr4	120534734	PDE5A	5.22E-08
rs1394608	chr5	155783294	SGCD	rs123491	chr4	23425990	MIR548AJ2	1.23E-08
rs10511467	chr9	7373051	.	rs10485193	chr6	117319747	.	4.40E-08
rs10511467	chr9	7373051	.	rs10489581	chr1	185121752	TRMT1L	1.31E-08
rs10511467	chr9	7373051	.	rs1046592	chr1	185069561	RNF2	1.31E-08
rs10511467	chr9	7373051	.	rs12046095	chr1	185107170	TRMT1L	1.31E-08
rs10511467	chr9	7373051	.	rs1394608	chr5	155783294	SGCD	3.41E-08
rs10492272	chr12	99265148	ANKS1B	rs7104698	chr11	36873565	.	5.40E-08
rs724211	chr12	72035610	ZFC3H1	rs3775652	chr4	143049980	INPP4B	3.87E-08
rs7104698	chr11	36873565	.	rs3775652	chr4	143049980	INPP4B	4.36E-08
rs7104698	chr11	36873565	.	rs657618	chr3	165406561	.	1.44E-08
rs7104698	chr11	36873565	.	rs475553	chr3	165376120	.	3.85E-09
rs10504040	chr8	41302667	.	rs1394608	chr5	155783294	SGCD	2.24E-08
rs2366248	chr7	79413489	.	rs1394608	chr5	155783294	SGCD	4.30E-08
rs1394608	chr5	155783294	SGCD	rs6847164	chr4	120534734	PDE5A	5.22E-08
rs1394608	chr5	155783294	SGCD	rs123491	chr4	23425990	MIR548AJ2	1.23E-08
rs9328536	chr9	134914385	MED27	rs1329428	chr1	196702810	CFH	9.93E-10
rs7467596	chr9	134912678	MED27	rs1329428	chr1	196702810	CFH	9.93E-10
rs10508731	chr10	28442788	MPP7	rs1329428	chr1	196702810	CFH	5.32E-09
rs10505107	chr8	108323384	ANGPT1	rs1329428	chr1	196702810	CFH	6.66E-09
rs324584	chr7	136650879	CHRM2,LOC349160	rs1329428	chr1	196702810	CFH	7.18E-09
rs324582	chr7	136650607	CHRM2,LOC349160	rs1329428	chr1	196702810	CFH	7.10E-09
rs10254116	chr7	33237489	BBS9	rs1329428	chr1	196702810	CFH	6.84E-09
rs3915771	chr5	13483969	.	rs1329428	chr1	196702810	CFH	8.20E-09
rs10489076	chr4	10270848	.	rs1329428	chr1	196702810	CFH	6.20E-09

rs4894367	chr3	138619560	.	rs1329428	chr1	196702810	CFH	2.52E-09
rs3922799	chr2	139758906	.	rs1329428	chr1	196702810	CFH	2.21E-09
rs4894367	chr3	138619560	.	rs380390	chr1	196701051	CFH	6.13E-10
