# Supplementary material for: AprioriGWAS, a New Pattern Mining Strategy for Detecting Genetic Variants Associated with Disease through Interaction Effects
Source: PLoS Comput Biol. 2014 Jun 5;10(6):e1003627. doi: 10.1371/journal.pcbi.1003627 (PMC4046917; doi:10.1371/journal.pcbi.1003627)
Supplement: Table S2 — Results of Bipolar Disorder from WTCCC. 200 pairs of variants show significant genotype pattern difference between case and control samples. (PDF) [file pcbi.1003627.s003.pdf]

Table S2 | Results of Bipolar Disorder from WTCCC

SNP_1	Chr.	Loc.	Gene	SNP_2	Chr.	Loc.	Gene	Nominal_p_value
rs17163470	chr1	26351382	EXTL1	rs4790667	chr17	1493816	SLC43A2	1.35E-10
rs571524	chr1	37303956	GRIK3	rs17755675	chr3	73712338	.	1.36E-08
rs571524	chr1	37303956	GRIK3	rs11714828	chr3	73727760	.	3.03E-08
rs569858	chr1	37304089	GRIK3	rs17755675	chr3	73712338	.	6.68E-09
rs569858	chr1	37304089	GRIK3	rs11714828	chr3	73727760	.	1.51E-08
rs527631	chr1	37309997	GRIK3	rs17755675	chr3	73712338	.	1.75E-08
rs547390	chr1	37317442	GRIK3	rs17755675	chr3	73712338	.	1.48E-08
rs17465176	chr1	59498111	.	rs9909476	chr17	80439733	NARF	2.35E-08
rs4915824	chr1	62797677	.	rs16909865	chr9	98207302	PTCH1	3.29E-08
rs11209478	chr1	69965181	.	rs12368906	chr12	94890011	.	2.24E-08
rs696666	chr1	75146093	.	rs8088821	chr18	4077059	DLGAP1	3.17E-08
rs11162341	chr1	77897713	AK5	rs6658302	chr1	77908985	AK5	1.59E-08
rs699768	chr1	116500096	.	rs1390395	chr3	88647887	.	1.66E-09
rs41691	chr7	120634636	CPED1	rs41694	chr7	120635798	CPED1	1.22E-09
rs4484634	chr7	158455089	NCAPG2	rs10266006	chr7	158474325	NCAPG2	6.20E-09
rs10253608	chr7	158469060	NCAPG2	rs10266006	chr7	158474325	NCAPG2	5.48E-10
rs12113120	chr7	158470080	NCAPG2	rs10266006	chr7	158474325	NCAPG2	6.46E-10
rs10949739	chr7	158472082	NCAPG2	rs10266006	chr7	158474325	NCAPG2	6.22E-10
rs4909259	chr7	158473900	NCAPG2	rs10266006	chr7	158474325	NCAPG2	5.45E-10
rs10266006	chr7	158474325	NCAPG2	rs10237585	chr7	158474373	NCAPG2	6.41E-10
rs10266006	chr7	158474325	NCAPG2	rs3793181	chr7	158481827	NCAPG2	9.89E-11
rs10266006	chr7	158474325	NCAPG2	rs6459895	chr7	158482870	NCAPG2	1.45E-09
rs10266006	chr7	158474325	NCAPG2	rs12698265	chr7	158489297	NCAPG2	6.40E-10
rs7825094	chr8	17468482	PDGFRL	rs2031532	chr13	50080847	PHF11	2.17E-08
rs2046223	chr8	26210178	PPP2R2A	rs17732285	chr8	92838980	.	1.66E-08

rs2139454	chr8	50259111	.	rs16907166	chr8	50264613	.	1.50E-08
rs10099080	chr8	54889118	TCEA1	rs11984645	chr8	55069305	.	5.05E-15
rs6473901	chr8	54918008	TCEA1	rs11984645	chr8	55069305	.	5.70E-16
rs6473903	chr8	54919891	TCEA1	rs11984645	chr8	55069305	.	1.07E-13
rs6983650	chr8	54937831	.	rs11984645	chr8	55069305	.	1.86E-16
rs8822	chr8	54960013	LYPLA1	rs11984645	chr8	55069305	.	3.37E-15
rs7844299	chr8	54962395	LYPLA1	rs11984645	chr8	55069305	.	1.84E-15
rs16919784	chr8	55063538	.	rs11984645	chr8	55069305	.	5.41E-13
rs11984645	chr8	55069305	.	rs4737503	chr8	55071319	.	4.99E-20
rs7001413	chr8	75010233	.	rs10973109	chr9	36861407	PAX5	5.54E-11
rs7832840	chr8	81136286	.	rs3784752	chr15	89453788	MFGE8	3.20E-08
rs2447183	chr8	120352978	.	rs2469997	chr8	120353267	.	6.40E-13
rs2469996	chr8	120353011	.	rs2469997	chr8	120353267	.	1.05E-12
rs2469997	chr8	120353267	.	rs2447179	chr8	120355775	.	1.98E-12
rs2469997	chr8	120353267	.	rs2447178	chr8	120356188	.	2.07E-12
rs2469997	chr8	120353267	.	rs2470002	chr8	120357424	.	1.08E-12
rs2469997	chr8	120353267	.	rs2470025	chr8	120364727	.	6.64E-13
rs2469997	chr8	120353267	.	rs2447169	chr8	120365063	.	4.29E-13
rs2469997	chr8	120353267	.	rs2470026	chr8	120365112	.	1.49E-13
rs2469997	chr8	120353267	.	rs2447168	chr8	120365613	.	1.36E-13
rs2469997	chr8	120353267	.	rs2470040	chr8	120385452	.	7.13E-13
rs11792896	chr9	73940190	.	rs2284372	chr20	1111874	PSMF1	1.47E-08
rs969668	chr9	106698319	.	rs4411335	chr12	129707854	TMEM132D	3.08E-08
rs969667	chr9	106698337	.	rs4411335	chr12	129707854	TMEM132D	4.53E-10
rs969667	chr9	106698337	.	rs2306515	chr12	129715859	TMEM132D	1.28E-09
rs1373247	chr9	106718321	.	rs4411335	chr12	129707854	TMEM132D	1.34E-09
rs1373247	chr9	106718321	.	rs2306515	chr12	129715859	TMEM132D	6.01E-09
rs1757200	chr10	18529835	CACNB2,CACNB2	rs8036241	chr15	90760289	SEMA4B	1.97E-09

rs1395043	chr10	59559737	.	rs1505923	chr10	59569726	.	1.17E-17
rs1505923	chr10	59569726	.	rs16911099	chr10	59572683	.	2.87E-17
rs7905405	chr10	61385195	.	rs2282031	chr14	90730943	PSMC1	1.14E-09
rs17459521	chr10	80496476	.	rs892542	chr10	80498057	.	3.28E-10
rs10160639	chr11	69999122	ANO1	rs1333245	chr13	94880876	GPC6	2.18E-08
rs10830589	chr11	90590660	.	rs577423	chr18	40522463	RIT2	2.45E-09
rs641024	chr11	134020848	JAM3	rs1950736	chr14	52600254	.	2.54E-08
rs641024	chr11	134020848	JAM3	rs2357442	chr14	52607967	.	1.69E-08
rs868169	chr11	114595001	.	rs1928510	chr13	102881745	FGF14	8.73E-09
rs2343866	chr12	26315082	.	rs2285166	chr22	44250630	SULT4A1	2.91E-08
rs7300697	chr12	26317170	.	rs2285166	chr22	44250630	SULT4A1	3.28E-08
rs10505993	chr12	26317799	.	rs2285166	chr22	44250630	SULT4A1	3.11E-08
rs10842686	chr12	26325379	.	rs2285166	chr22	44250630	SULT4A1	3.25E-08
rs10842688	chr12	26338294	.	rs2285166	chr22	44250630	SULT4A1	2.42E-08
rs10842689	chr12	26338365	.	rs2285166	chr22	44250630	SULT4A1	2.66E-08
rs11168985	chr12	39045983	.	rs826886	chr12	39095797	CPNE8	1.65E-10
rs11168985	chr12	39045983	.	rs826838	chr12	39106731	CPNE8	1.19E-08
rs2241960	chr12	46580618	SLC38A1	rs10521320	chr16	55576161	LPCAT2	1.81E-08
rs7325920	chr13	24711395	.	rs7139605	chr13	96811028	HS6ST3	7.72E-09
rs9561326	chr13	94007107	GPC6	rs9561329	chr13	94011169	GPC6	2.08E-11
rs11624794	chr14	24260146	.	rs1958305	chr14	24273124	.	8.20E-17
rs1958305	chr14	24273124	.	rs17184408	chr14	24282020	.	1.74E-14
rs1439170	chr14	46638877	LINC00871	rs7152370	chr14	46735956	LINC00871	9.85E-14
rs858870	chr14	46666812	LINC00871	rs7152370	chr14	46735956	LINC00871	4.61E-10
rs17737767	chr14	46718257	LINC00871	rs7152370	chr14	46735956	LINC00871	1.05E-13
rs7152370	chr14	46735956	LINC00871	rs10483596	chr14	46740909	LINC00871	1.61E-16
rs7152370	chr14	46735956	LINC00871	rs8017858	chr14	46741726	LINC00871	2.76E-12
rs17105918	chr14	72494808	RGS6	rs4902976	chr14	72505876	RGS6	8.56E-12

rs7162070	chr15	39920918	FSIP1	rs16969478	chr15	39930953	FSIP1	4.67E-09
rs1876853	chr15	39921167	FSIP1	rs16969478	chr15	39930953	FSIP1	5.52E-09
rs8029602	chr15	39930540	FSIP1	rs16969478	chr15	39930953	FSIP1	2.03E-08
rs16969475	chr15	39930869	FSIP1	rs16969478	chr15	39930953	FSIP1	1.67E-08
rs746655	chr15	74107677	.	rs921535	chr15	74111343	.	8.18E-15
rs886889	chr17	12804201	ARHGAP44	rs10521202	chr17	12814564	ARHGAP44	5.80E-17
rs10521202	chr17	12814564	ARHGAP44	rs5017214	chr17	12817783	ARHGAP44	1.28E-12
rs12941700	chr17	30185022	COPRS	rs9906443	chr17	30185565	COPRS	6.87E-11
rs12941700	chr17	30185022	COPRS	rs9899093	chr17	30191954	UTP6	6.70E-11
rs12941700	chr17	30185022	COPRS	rs7209493	chr17	30197323	UTP6	4.28E-09
rs12941700	chr17	30185022	COPRS	rs1034626	chr17	30219500	UTP6	3.16E-08
rs11570451	chr17	45266163	CDC27	rs11570441	chr17	45267985	.	1.09E-12
rs17765129	chr17	69039331	.	rs2193346	chr17	69040230	.	4.76E-12
rs1944328	chr18	61838457	LOC284294	rs1944327	chr18	61838947	LOC284294	5.30E-25
rs1944328	chr18	61838457	LOC284294	rs8091006	chr18	61842934	LOC284294	8.18E-28
rs1944328	chr18	61838457	LOC284294	rs9675798	chr18	61856967	LOC284294	3.45E-27
rs1944328	chr18	61838457	LOC284294	rs9676116	chr18	61857065	LOC284294	2.13E-27
rs6681017	chr1	181508326	CACNA1E	rs709425	chr16	12339045	SNX29	3.05E-08
rs4657934	chr1	194702235	.	rs2094278	chr13	51728207	LINC00371	3.35E-08
rs10922404	chr1	198321848	.	rs7901171	chr10	3905883	.	2.90E-08
rs16847947	chr1	201150096	.	rs7115118	chr11	71285221	.	2.46E-08
rs4362037	chr1	202662356	SYT2	rs4621318	chr3	186921468	.	8.69E-09
rs4844637	chr1	208262224	PLXNA2	rs10863695	chr1	208263657	PLXNA2	1.63E-11
rs11118748	chr1	221715136	.	rs12136041	chr1	221718224	.	2.53E-11
rs4305308	chr2	1148426	SNTG2	rs10512617	chr17	76693551	CYTH1	1.24E-08
rs4305308	chr2	1148426	SNTG2	rs17736494	chr17	76728668	CYTH1	9.51E-09
rs306184	chr2	6646177	.	rs7624992	chr3	3233271	.	9.61E-09
rs12052774	chr2	18902708	.	rs2493243	chr6	156939117	.	4.62E-09

rs8130402	chr21	40420409	.	rs999789	chr21	40426451	.	4.82E-17
rs2836860	chr21	40423964	.	rs999789	chr21	40426451	.	9.62E-11
rs4279007	chr21	40424954	.	rs999789	chr21	40426451	.	9.69E-11
rs999789	chr21	40426451	.	rs999790	chr21	40426768	.	1.78E-11
rs999789	chr21	40426451	.	rs428424	chr21	40445319	.	5.92E-11
rs999789	chr21	40426451	.	rs445593	chr21	40448382	.	5.11E-11
rs350747	chr2	52868656	.	rs350753	chr2	52873788	.	9.51E-11
rs17046061	chr2	54864030	SPTBN1	rs17046067	chr2	54867496	SPTBN1	2.35E-12
rs17013615	chr2	77241510	LRRTM4	rs371657	chr19	46937731	.	2.47E-08
rs12997174	chr2	80373814	CTNNA2	rs7859432	chr9	1321394	.	3.43E-08
rs4641957	chr2	105746037	.	rs732568	chr7	120560714	.	8.62E-09
rs11894003	chr2	133774800	NCKAP5	rs4740158	chr9	133325472	ASS1	6.23E-09
rs3113224	chr2	138762056	HNMT	rs12912140	chr15	87216164	AGBL1	2.02E-08
rs6735884	chr2	154786777	GALNT13	rs17344410	chr3	65104376	.	2.50E-08
rs12693918	chr2	154961754	GALNT13	rs17344410	chr3	65104376	.	2.65E-08
rs1961474	chr2	154963177	GALNT13	rs17344410	chr3	65104376	.	2.81E-08
rs1020732	chr2	161144239	RBMS1	rs12692590	chr2	161153197	RBMS1	9.04E-11
rs16866984	chr2	180630846	ZNF385B	rs4077322	chr8	108305555	ANGPT1	2.31E-08
rs16866984	chr2	180630846	ZNF385B	rs4133396	chr8	108314797	ANGPT1	2.98E-08
rs16849921	chr2	214061022	.	rs10197379	chr2	214061239	.	1.19E-31
rs16849921	chr2	214061022	.	rs12694298	chr2	214061907	.	3.22E-30
rs2356656	chr2	192381934	.	rs10512067	chr9	80711471	.	1.30E-08
rs2356656	chr2	192381934	.	rs10870047	chr9	80718738	.	1.77E-08
rs2356656	chr2	192381934	.	rs10870048	chr9	80718792	.	1.14E-08
rs2035219	chr3	8940417	RAD18	rs279937	chr13	103704077	SLC10A2	1.15E-09
rs1992348	chr3	34086193	.	rs6469617	chr8	117038175	LINC00536	2.57E-08
rs10510819	chr3	59691572	.	rs4299909	chr7	30969120	.	7.77E-11
rs1114002	chr3	145071574	.	rs9633957	chr11	124326024	.	3.22E-08

rs9290197	chr3	162745208	.	rs6806447	chr3	162762902	.	8.05E-12
rs9290197	chr3	162745208	.	rs4858916	chr3	162764257	.	4.99E-12
rs9290197	chr3	162745208	.	rs11570441	chr17	45267985	.	5.54E-13
rs6767600	chr3	171506951	PLD1	rs2879526	chr18	50769543	DCC	2.67E-08
rs4600860	chr4	5508488	.	rs4688938	chr4	5516030	.	2.68E-11
rs4688938	chr4	5516030	.	rs4586871	chr4	5516378	.	5.78E-15
rs4688938	chr4	5516030	.	rs2267654	chr4	5527249	C4orf6	3.22E-11
rs4688938	chr4	5516030	.	rs16837389	chr4	5536310	.	9.49E-11
rs560164	chr4	13073411	.	rs17693758	chr5	4726710	.	8.57E-09
rs2132631	chr4	16653326	LDB2	rs17463021	chr10	80693615	.	1.96E-08
rs1948584	chr4	44909853	.	rs10032741	chr4	44960444	.	7.42E-10
rs7690764	chr4	44924382	.	rs10032741	chr4	44960444	.	6.55E-09
rs1845945	chr4	44933047	.	rs10032741	chr4	44960444	.	3.14E-09
rs9998147	chr4	44934205	.	rs10032741	chr4	44960444	.	1.30E-09
rs10032741	chr4	44960444	.	rs1908806	chr4	44964953	.	6.37E-11
rs6532650	chr4	97685777	.	rs9667214	chr11	78821945	TENM4	6.24E-10
rs444601	chr5	14007930	.	rs413198	chr5	14008995	.	4.01E-10
rs7735940	chr5	36423931	.	rs12522034	chr5	36425593	.	3.67E-09
rs7735940	chr5	36423931	.	rs12515142	chr5	36426411	.	1.40E-11
rs270586	chr5	38072705	.	rs10898818	chr11	69820567	.	3.29E-08
rs2935260	chr5	54444880	CDC20B	rs2992406	chr5	54444983	CDC20B	1.78E-13
rs10805849	chr5	87304268	.	rs881601	chr5	87313537	.	1.58E-10
rs4916819	chr5	87313273	.	rs881601	chr5	87313537	.	1.80E-10
rs17486181	chr5	96234683	ERAP2	rs17486915	chr5	96244719	ERAP2	3.14E-08
rs17486915	chr5	96244719	ERAP2	rs17628359	chr5	96275961	LNPEP	2.22E-08
rs12515561	chr5	152955611	GRIA1	rs1552835	chr5	152959769	GRIA1	2.22E-11
rs12515563	chr5	152955632	GRIA1	rs1552835	chr5	152959769	GRIA1	2.63E-10
rs12515520	chr5	152955663	GRIA1	rs1552835	chr5	152959769	GRIA1	3.40E-11

rs1552837	chr5	152959669	GRIA1	rs1552835	chr5	152959769	GRIA1	5.90E-11
rs1552835	chr5	152959769	GRIA1	rs17519558	chr5	152960319	GRIA1	7.64E-12
rs1552835	chr5	152959769	GRIA1	rs17519656	chr5	152960528	GRIA1	1.11E-11
rs1552835	chr5	152959769	GRIA1	rs17591636	chr5	152960615	GRIA1	2.57E-11
rs13163917	chr5	157832300	.	rs1414770	chr10	83987906	NRG3	2.83E-08
rs153300	chr5	160726532	GABRB2	rs252978	chr5	160730568	GABRB2	9.18E-09
rs252982	chr5	160728170	GABRB2	rs252978	chr5	160730568	GABRB2	8.95E-09
rs252978	chr5	160730568	GABRB2	rs252977	chr5	160730639	GABRB2	4.13E-11
rs17069979	chr5	167624108	TENM2	rs4298516	chr8	135283869	.	1.05E-08
rs2173018	chr5	167624416	TENM2	rs4298516	chr8	135283869	.	1.18E-08
rs2438074	chr6	1269690	.	rs2438083	chr6	1277371	.	8.18E-09
rs9357438	chr6	9492158	.	rs9357440	chr6	9492393	.	2.90E-17
rs450008	chr6	40495844	LRFN2	rs1819287	chr10	97275646	SORBS1	8.66E-10
rs450008	chr6	40495844	LRFN2	rs4498913	chr10	97276492	SORBS1	8.33E-10
rs450008	chr6	40495844	LRFN2	rs6584006	chr10	97276810	SORBS1	2.13E-09
rs450008	chr6	40495844	LRFN2	rs10736089	chr10	97279234	SORBS1	2.68E-09
rs369306	chr6	40496106	LRFN2	rs1819287	chr10	97275646	SORBS1	1.01E-09
rs369306	chr6	40496106	LRFN2	rs4498913	chr10	97276492	SORBS1	9.94E-10
rs369306	chr6	40496106	LRFN2	rs6584006	chr10	97276810	SORBS1	2.60E-09
rs369306	chr6	40496106	LRFN2	rs10736089	chr10	97279234	SORBS1	3.19E-09
rs6905412	chr6	95896515	.	rs6910024	chr6	95896731	.	5.68E-10
rs6905412	chr6	95896515	.	rs1319912	chr6	95918495	.	3.76E-16
rs6905412	chr6	95896515	.	rs6928585	chr6	95948860	.	1.84E-10
rs6905412	chr6	95896515	.	rs4579361	chr6	96083854	.	2.21E-11
rs2236014	chr6	153310332	MTRF1L	rs2236016	chr6	153313460	MTRF1L	9.20E-15
rs2236014	chr6	153310332	MTRF1L	rs9397578	chr6	153329508	.	4.08E-15
rs9384465	chr6	156731222	.	rs3779536	chr7	127233977	FSCN3	2.10E-09
rs9365945	chr6	166455280	.	rs2017393	chr9	112820600	AKAP2,PALM2	2.50E-08

rs12699827	chr7	17135544	.	rs6983436	chr8	104956705	RIMS2	1.79E-08
rs985307	chr7	19573763	.	rs985882	chr7	19575505	.	5.31E-11
rs985306	chr7	19574066	.	rs985882	chr7	19575505	.	9.73E-11
rs2192481	chr7	19574677	.	rs985882	chr7	19575505	.	2.90E-10
rs985882	chr7	19575505	.	rs985881	chr7	19575555	.	1.14E-11
rs7781714	chr7	23593479	.	rs6949019	chr7	23594050	.	5.30E-11
rs17689617	chr7	41014524	.	rs8105122	chr19	16089378	.	2.70E-08
